# Stimulation of the muscarinic receptor M4 regulates neural precursor cell proliferation and promotes adult hippocampal neurogenesis

**DOI:** 10.1242/dev.201835

**Published:** 2024-01-02

**Authors:** Lidia I. Madrid, Katelyn Hafey, Saurabh Bandhavkar, Gabriela O. Bodea, Javier Jimenez-Martin, Michael Milne, Tara L. Walker, Geoffrey J. Faulkner, Elizabeth J. Coulson, Dhanisha J. Jhaveri

**Affiliations:** ^1^ Queensland Brain Institute, The University of Queensland, Brisbane QLD 4072, Queensland, Australia; ^2^ Mater Research Institute - The University of Queensland, Translational Research Institute, Brisbane QLD 4102, Queensland, Australia; ^3^ School of Biomedical Sciences, The University of Queensland, Brisbane QLD 4072, Queensland, Australia

**Keywords:** Adult neurogenesis, Neural precursor cells, Hippocampus, Acetylcholine, Cholinergic receptors, Medial septum

## Abstract

Cholinergic signaling plays a crucial role in the regulation of adult hippocampal neurogenesis; however, the mechanisms by which acetylcholine mediates neurogenic effects are not completely understood. Here, we report the expression of muscarinic acetylcholine receptor subtype M4 (M4 mAChR) on a subpopulation of neural precursor cells (NPCs) in the adult mouse hippocampus, and demonstrate that its pharmacological stimulation promotes their proliferation, thereby enhancing the production of new neurons *in vivo*. Using a targeted ablation approach, we also show that medial septum (MS) and the diagonal band of Broca (DBB) cholinergic neurons support both the survival and morphological maturation of adult-born neurons in the mouse hippocampus. Although the systemic administration of an M4-selective allosteric potentiator fails to fully rescue the MS/DBB cholinergic lesion-induced decrease in hippocampal neurogenesis, it further exacerbates the impairment in the morphological maturation of adult-born neurons. Collectively, these findings reveal stage-specific roles of M4 mAChRs in regulating adult hippocampal neurogenesis, uncoupling their positive role in enhancing the production of new neurons from the M4-induced inhibition of their morphological maturation, at least in the context of cholinergic signaling dysfunction.

## Teaser

**Summary:** The muscarinic acetylcholine receptor subtype M4 (M4 mAChR) is expressed in a subpopulation of neural precursor cells in the adult mouse hippocampus, where is plays a stage-specific role in neurogenesis.

## INTRODUCTION

The hippocampus is a key brain region in which the developmental program of neurogenesis remains functional during adult life (Ming and Song, 2011). The heightened plasticity of these adult-born neurons has been shown to impact neuronal activity within the local circuitry and to contribute to the regulation of select cognitive and mood-related functions in rodents (Christian et al., 2014). The process of adult hippocampal neurogenesis, which encompasses the proliferation of resident neural precursor cells (NPCs), their differentiation into neurons and their integration into the existing circuitry, is sensitive to neural activity-mediated regulation (Jhaveri et al., 2012; Ming and Song, 2011). Among a range of intrinsic and extrinsic factors, several neurotransmitters, including glutamate, γ-aminobutyric acid (GABA), serotonin, norepinephrine and dopamine, have been shown to regulate various stages of adult neurogenesis, serving both non-synaptic trophic and synaptic roles (Berg et al., 2013; Jhaveri et al., 2010, 2015; Song et al., 2012).

The neurotransmitter acetylcholine has also been linked to the regulation of adult hippocampal neurogenesis, as well as hippocampus-dependent cognitive processes (Cooper-Kuhn et al., 2004; Madrid et al., 2021; Mohapel et al., 2005). The dentate gyrus, which harbors populations of quiescent and active NPCs (Bonaguidi et al., 2011; Jhaveri et al., 2010; Lugert et al., 2010; Walker et al., 2008), is richly innervated by cholinergic fibers that originate from the cell bodies residing in the medial septum (MS) and the diagonal band of Broca (DBB) nuclei of the basal forebrain cholinergic system (Kaneko et al., 2006). Notably, retrograde tracing experiments in animals have shown that, during their immature phase, adult-born hippocampal neurons receive monosynaptic innervation from the MS cholinergic neurons (Deshpande et al., 2013; Vivar et al., 2012).

Given that the loss of basal forebrain cholinergic neurons, including those that innervate the hippocampus (Kerbler et al., 2013; Schmitz and Nathan Spreng, 2016), and a progressive decline in hippocampal neurogenesis (Moreno-Jiménez et al., 2019) are among the early pathogenic events that correlate with the cognitive impairments observed in Alzheimer's disease, a number of studies have explored the link between cholinergic signaling and adult hippocampal neurogenesis in animal models. Supporting the neurogenic role of septal cholinergic innervation, global lesion of the basal forebrain cholinergic neurons in rats has been shown to impair hippocampal neurogenesis by reducing the survival of newborn neurons (Cooper-Kuhn et al., 2004; Mohapel et al., 2005). Corroborating these findings, the inhibition of cholinergic activity using the muscarinic antagonist scopolamine has been found to decrease cell proliferation in the adult hippocampus (Chen et al., 2018), whereas intracerebroventricular infusions of oxotremorine, a selective muscarinic receptor agonist, led to an increase in proliferation (Cooper-Kuhn et al., 2004; Mohapel et al., 2005; van Kampen and Eckman, 2010). Interestingly, the acetylcholinesterase inhibitor donepezil, which is clinically used to treat cognitive deficits in Alzheimer's disease, has been shown to enhance hippocampal neurogenesis (Kotani et al., 2006).

Although global basal forebrain cholinergic lesions and pharmacological approaches have suggested an important role for cholinergic signaling in regulating adult hippocampal neurogenesis (Cooper-Kuhn et al., 2004; Mohapel et al., 2005), and immunohistochemical analysis has revealed the expression of M1 muscarinic acetylcholine receptors (mAChRs) and M4 mAChRs on a subpopulation of proliferating cells (Mohapel et al., 2005), the mechanism(s) by which acetylcholine mediates neurogenic effects are only now being elucidated. For example, it has recently been reported that astrocytic M1 mAChRs modulate hippocampal neurogenesis by regulating brain-derived neurotrophic factor signaling (Li et al., 2022), and nestin-positive precursors express M1 mAChRs that, when stimulated, promote proliferation (Itou et al., 2011; Shin et al., 2015).

Here, we report the expression of M4 mAChRs on a subpopulation of hippocampal precursor cells and demonstrate the effect of manipulating M4 receptor activity on various stages of adult neurogenesis, including the proliferation and differentiation of NPCs, as well as the maturation of newborn neurons, under baseline conditions as well as after cholinergic neuron loss.

## RESULTS

### A subpopulation of hippocampal NPCs expresses M4 mAChRs

**Fig. 1.**
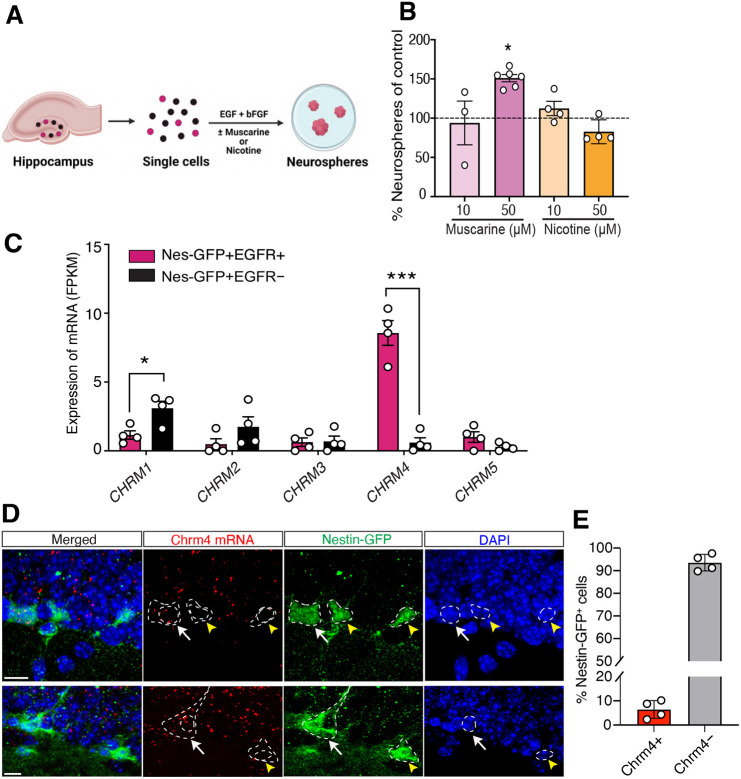
**M4 mAChRs are expressed by a subpopulation of hippocampal NPCs.** (A) Schematic of the neurosphere assay used to assess NPC activity from the hippocampus. (B) Treatment of adult hippocampal cells with muscarine (50 μM) but not nicotine generated significantly more neurospheres than were produced in the control medium *P<0.05; paired Student's *t*-test). (C) RNA-seq data showing expression of all muscarinic receptors (*CHRM1-CHRM5*) in hippocampal NPCs (Nestin-GFP^+^/EGFR^+^) and non-NPC (Nestin-GFP^−^/EGFR^−^) populations. *CHRM4* is enriched in the hippocampal NPCs, whereas *CHRM1* is enriched in Nestin-GFP^+^/EGFR^—^ cells (*P<0.05, ***P<0.001; paired Student's *t*-test). FPKM, fragments per kilobase of transcript per million mapped reads. (D) Representative confocal images showing Chrm4 transcripts (red) detected by RNA fluorescent *in situ* hybridization, Nestin-GFP immunohistochemistry (green) and DAPI staining for nuclei (blue) in the dentate gyrus. Dashed lines outline nuclear and cellular boundaries defined by DAPI and Nestin-GFP. Chrm4 transcripts are present in a subpopulation of Nestin-GFP cells (arrows), including the Nestin-GFP^+^ cells exhibiting RGL morphology. Arrowheads indicate Nestin-GFP^+^ cells with no Chrm4 transcripts. Scale bars: 10 μm. (E) Quantification of the proportion of Nestin-GFP^+^ cells containing Chrm4 transcripts. Data are mean±s.e.m.

The neurogenic niche in the dentate gyrus is richly innervated by cholinergic fibers originating from the cell bodies residing in the MS/DBB, and fibers containing choline acetyltransferase, the enzyme responsible for acetylcholine synthesis, have been observed in close proximity to NPCs (Kaneko et al., 2006). Although a rapid rise in intracellular calcium levels in NPCs has been reported after the application of acetylcholine to hippocampal slices (Itou et al., 2011), whether acetylcholine directly regulates the activity, i.e. proliferation of distinct subpopulations of hippocampal NPCs (Jhaveri et al., 2015), is currently unknown. To address this, we first stimulated murine hippocampal NPCs with drugs targeting the two major classes of acetylcholine receptors, i.e. metabotropic muscarinic and ionotropic nicotinic receptors, and assayed their proliferative activity using the neurosphere assay (Fig. 1A). As the neurospheres were generated by clonal expansion and proliferation of an individual NPC, a change in their numbers in response to cholinergic stimulation reflected the number of proliferating NPCs. A single-cell suspension of primary hippocampal cells was treated with or without muscarine or nicotine in the presence of the growth factors epidermal growth factor (EGF) and basic fibroblast growth factor (bFGF; control condition). Treatment with 50 µM muscarine but not nicotine led to a significant increase in the number of neurospheres compared with the control condition, indicating that mAChRs may regulate the activity of hippocampal NPCs (Fig. 1B).

We next sought to identify the cholinergic receptor(s) expressed by NPCs and examine the effects of stimulating muscarinic receptor(s) on NPC activity. We have previously identified and purified hippocampal NPCs using concomitant selection of Nestin-GFP^+^ and epidermal growth factor receptor-expressing (EGFR^+^) cells, which constitute 4.7±1.0% of the total Nestin-GFP cells (Jhaveri et al., 2015). RNA-seq analysis of this population revealed selective expression and enrichment of *CHRM4* (the gene encoding M4 mAChRs) but not *CHRM1*, *CHRM2*, *CHRM3* or *CHRM5*, in Nestin-GFP^+^EGFR^+^ hippocampal NPCs compared with Nestin-GFP^+^EGFR^−^ cells (Fig. 1C). Of note, *CHRM1* was expressed in Nestin-GFP^+^ cells, as reported previously (Shin et al., 2015); however, the expression was significantly enriched in the Nestin-GFP^+^EGFR^−^ compared with the Nestin-GFP^+^EGFR^+^ fraction, which harbors the majority of neurosphere-forming NPCs (Jhaveri et al., 2015). We also confirmed the presence of Chrm4 transcripts in a subpopulation of Nestin-GFP^+^ cells using single-molecule *in situ* hybridization (Fig. 1D) and found that 6.4±1.8% of the total Nestin-GFP^+^ cells contained Chrm4 transcripts (Fig. 1E).

### Pharmacological stimulation of M4 mAChRs promotes proliferation of a subpopulation of hippocampal NPCs

**Fig. 2.**
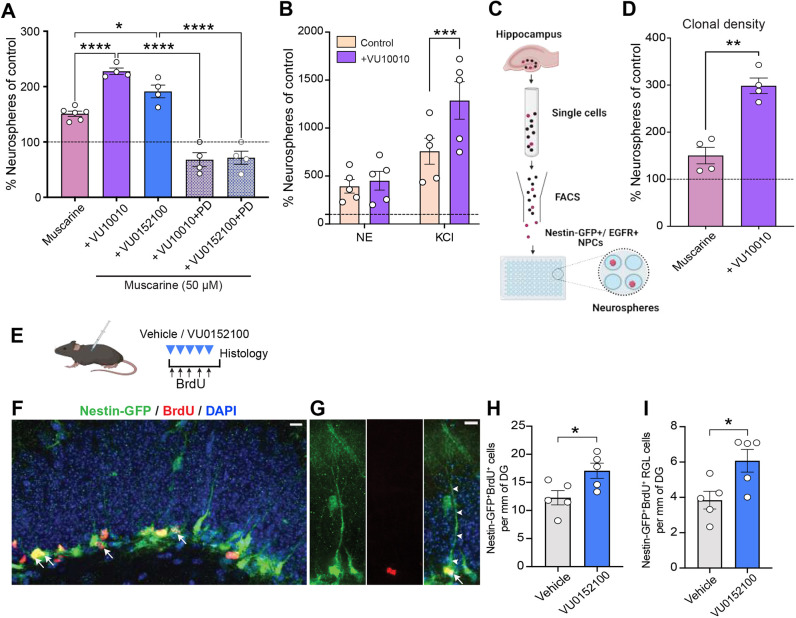
**Pharmacological stimulation of M4 mAChRs enhances the proliferation of a subpopulation of hippocampal NPCs *ex vivo* and *in vivo*.** (A) Treatment with the M4 mAChR-selective allosteric potentiators VU10010 (10 μM) and VU0152100 (10 μM) in the presence of muscarine (50 μM) led to a twofold increase in the number of neurospheres compared with the control (shown as a dotted line). PD102807 (PD; 1 μM), a selective M4 mAChR antagonist completely blocked this VU10010 and VU0152100-mediated increase in neurosphere formation (one-way ANOVA). (B) Relative number of neurospheres (compared with the control; shown as a dotted line) obtained in norepinephrine (NE) and KCl treatment conditions in the absence or presence of VU10010 (10 μM). VU10010 significantly increased the number of neurospheres in KCl- but not NE-treated conditions (two-way ANOVA). (C) Schematic showing the experimental design for the purification of Nestin-GFP^+^/EGFR^+^ NPCs from the adult hippocampus using FACS and an assay to assess their proliferative capacity at clonal density. (D) In the clonal density assay, the treatment of Nestin-GFP^+^/EGFR^+^ NPCs with VU10010 (10 μM) directly potentiated the effects of muscarine, with the relative number of neurospheres obtained in the presence of muscarine+VU10010 being significantly higher than that in muscarine alone (paired Student's *t*-test). (E) Experimental timeline for the systemic administration of VU0152100 to examine hippocampal NPC proliferation. (F,G) Confocal images showing labeling for Nestin-GFP (green), BrdU (red) and DAPI (blue) in the dentate gyrus of the hippocampus. Arrows indicate proliferating Nestin-GFP^+^BrdU^+^ NPCs. (G) An example of a proliferating (BrdU^+^) Nestin-GFP^+^ RGL morphology (arrowheads indicate the process). (H,I) Quantification of BrdU incorporation in Nestin-GFP^+^ cells (H) and Nestin-GFP^+^BrdU^+^ cells (I) with RGL morphology in VU0152100-treated mice compared with vehicle-treated mice (unpaired Student's *t*-test). DG, dentate gyrus. Scale bars: 10 μm. Data are mean±s.e.m. *P<0.05, **P<0.01, ***P<0.001, ****P<0.0001.

Having identified that M4 mAChRs were preferentially expressed in the Nestin-GFP^+^EGFR^+^ hippocampal NPC population, we next examined the effects of pharmacological agents that selectively stimulate (VU10010 and VU0152100) or inhibit (PD102807) their activity in NPC cultures. As observed earlier, treatment of NPCs with muscarine alone led to a significant increase in the number of neurospheres, with the M4 mAChR-specific allosteric modulators VU10010 or VU0152100 further potentiating this effect (Fig. 2A). However, in the presence of PD102807, a selective M4 mAChR antagonist, this increase was completely abrogated, thereby establishing the specificity of the response (Fig. 2A). These findings suggest that cholinergic signaling via M4 mAChRs can regulate the proliferative activity of hippocampal NPCs *ex vivo*.

We have previously demonstrated the presence of distinct subpopulations of activatable, self-renewing and multipotent NPCs that are responsive to either KCl or norepinephrine (Jhaveri et al., 2010; Walker et al., 2008). As our studies have found that these NPC subpopulations are not only differentially distributed along the septotemporal axis of the adult hippocampus but are also differentially regulated by neurogenic modulators such as GABA, corticosterone and selenium (Jhaveri et al., 2015; Leiter et al., 2022), we further investigated whether stimulation of M4 mAChRs activated either or both of these populations (Fig. 2B). Treatment with KCl+VU10010 resulted in a significant increase in the number of neurospheres compared with KCl treatment alone, whereas no such additive effect was observed in the presence of norepinephrine, suggesting that stimulation of M4 mAChRs promotes the proliferation of the same subpopulation of hippocampal NPCs as that which is responsive to norepinephrine.

To probe the direct effects of stimulating M4 mAChRs (in the absence of any niche cells) on the regulation of hippocampal NPC proliferation and subsequent neurosphere formation, hippocampal NPCs (Nestin-GFP^+^/EGFR^+^) were isolated using flow cytometry and plated at clonal density (<1 cell per well; Fig. 2C). At this density, treatment with VU10010 in the presence of muscarine still led to a significant increase in the number of neurospheres compared with the control (Fig. 2D). These findings indicate that cholinergic signaling via M4 mAChRs directly promotes the activity of a subpopulation of hippocampal NPCs *ex vivo*.

Finally, to assess whether M4 mAChR stimulation promotes the proliferation of hippocampal NPCs *in vivo*, Nestin-GFP mice were administered the blood-brain barrier-permeable M4 mAChR potentiator VU0152100 systemically via intraperitoneal (i.p.) injections daily for 5 days (Fig. 2E); the incorporation of 5-bromo-2-deoxyuridine (BrdU) in Nestin-GFP^+^ NPCs, including those exhibiting radial glial-like (RGL) morphology, was evaluated (Fig. 2F,G). Stereological quantification showed a significant increase in the total number of Nestin-GFP^+^BrdU^+^ NPCs (Fig. 2H) and the number of RGL Nestin-GFP^+^BrdU^+^ cells (Fig. 2I) in the VU0152100-treated compared with the vehicle-treated mice, providing evidence for enhanced proliferation of NPCs *in vivo* after stimulation of M4 mAChRs.

### Stimulation of M4 mAChRs enhances adult hippocampal neurogenesis *in vivo*

**Fig. 3.**
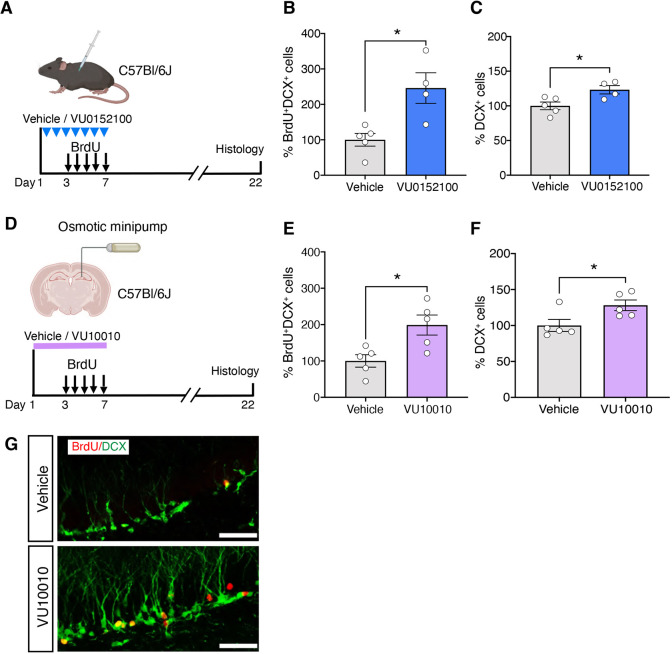
**Stimulation of M4 mAChRs enhances adult hippocampal neurogenesis *in vivo.*** (A) Experimental timeline for the systemic administration of VU0152100 to analyze the production of new neurons in the hippocampus. (B,C) The numbers of newborn neurons (B; BrdU^+^DCX^+^) and total immature neurons (C; DCX^+^) were significantly higher in the VU0152100-treated mice. (D) Experimental timeline for the unilateral infusion of VU10010 into the hippocampus. (E,F) Quantification showing a significant increase in the number of newborn neurons (E; BrdU^+^DCX^+^) and total immature neurons (F; DCX^+^) in the VU10010- versus vehicle-infused hippocampus. (G) Representative confocal images of the dentate gyrus showing BrdU^+^ (red) and DCX^+^ (green) cells in the vehicle- and VU10010-treated mice. Scale bars: 50 µm. Data are mean±s.e.m.: *P<0.05; unpaired Student's *t*-test.

We next examined whether the stimulation of NPCs with selective M4 mAChR allosteric potentiators leads to an enhancement in the production of new neurons *in vivo*. Mice were administered VU0152100 systemically via i.p. injections daily for 7 days (Fig. 3A). To label the proliferating cells, BrdU was administered daily for a period of 5 days, starting 48 h after the VU0152100 administration, and brains were collected 15 days after the end of treatment. Subsequent analysis revealed a significant increase in the number of newly generated neurons, identified as those labeled with BrdU and expressing the immature neuron marker doublecortin (BrdU^+^DCX^+^), as well as the total number of immature neurons (DCX^+^) in the VU0152100- compared with the vehicle-treated mice (Fig. 3B,C).

To further corroborate these findings, VU10010, which is blood-brain barrier impermeable, or vehicle was infused directly into the hippocampus over a period of 7 days via a unilateral cannula (Fig. 3D). Similar to the results obtained following the VU0152100 treatment, significant increases in the number of BrdU^+^DCX^+^ cells as well as the total number of DCX^+^ cells, were observed in the VU10010-infused mice compared with the controls (Fig. 3E-G). Collectively, these data demonstrate that pharmacological stimulation of M4 mAChRs *in vivo* enhances the production and/or survival of new neurons, boosting the overall level of adult hippocampal neurogenesis.

### Loss of MS/DBB cholinergic neurons reduces the survival and impairs the morphological maturation of newborn neurons in the hippocampus

**Fig. 4.**
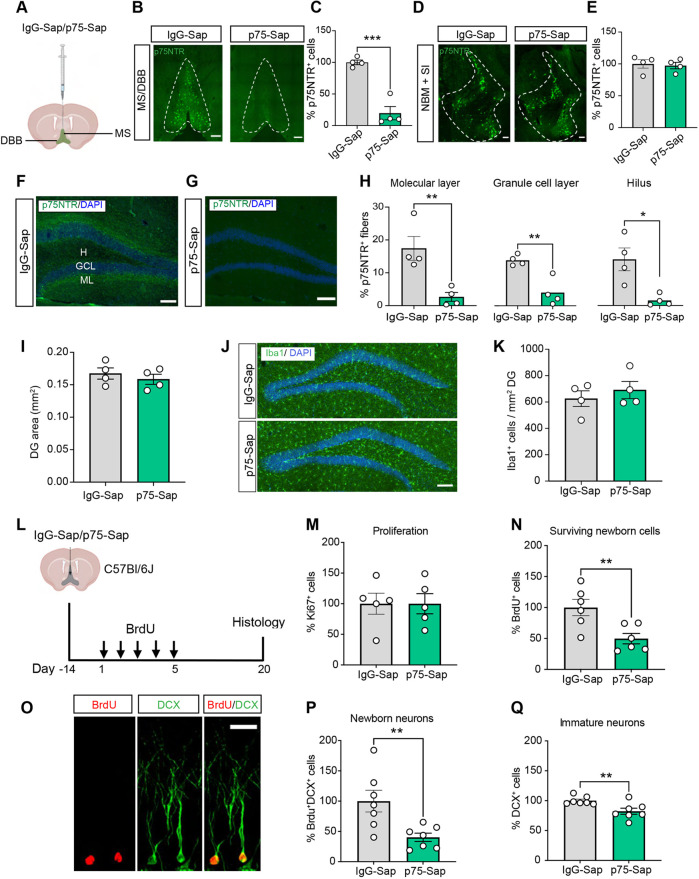
**Selective MS/DBB cholinergic lesion leads to impairments in adult hippocampal neurogenesis.** (A) Experimental timeline showing selective ablation of MS/DBB cholinergic neurons in the mouse brain by infusion of p75-Sap. Control mice were infused with IgG-Sap. (B) Representative images displaying loss of p75NTR^+^ (green) cholinergic neurons in the MS/DBB of p75-Sap-treated mice compared with control mice. (C) Quantification showing ablation of p75NTR^+^ cholinergic neurons in the MS/DBB in p75-Sap- compared with IgG-Sap-infused mice. (D) Representative images showing p75NTR^+^ neurons in the NBM and SI. (E) No loss of NBM+SI cholinergic neurons was observed in p75-Sap-treated compared with the IgG-Sap-treated mice. (F,G) A confocal image displaying p75NTR^+^ fibers (green) and nuclei stained with DAPI (blue) in the hippocampus in IgG-Sap-treated (F) and p75-Sap-treated (G) mice. (H) Quantification of p75NTR^+^ fibers in the molecular layer (ML), granule cell layer (GCL) and hilus (H), revealing their profound loss in p75-Sap-infused mice. (I) Quantification of dentate gyrus area. (J) Representative images showing Iba1^+^ (green) microglia in the hippocampus of IgG-Sap and p75-Sap-infused mice. Nuclei are stained with DAPI (blue). (K) Quantification of the number of Iba1^+^ microglia in the dentate gyrus. (L) Overview of the experimental timeline to evaluate adult neurogenesis. Two weeks after IgG-Sap or p75-Sap infusion in the MS/DBB, mice received daily injections of BrdU for 5 days and were sacrificed 3 weeks later. (M,N,P,Q) Quantification represented as ‘percentage change’ relative to IgG-Sap-infused controls. (M) No difference in the total number of proliferating cells (Ki67^+^) was observed between the groups. (N) There was a significant reduction in the total number of BrdU^+^ cells in p75-Sap-treated mice compared with controls. (O) A confocal image showing newborn hippocampal neurons co-labeled with BrdU (red) and DCX (green). (P) Quantification of newly generated immature neurons (BrdU^+^DCX^+^) and (Q) total immature neurons (DCX^+^) in IgG-Sap- and p75-Sap-infused mice. DG, dentate gyrus. Scale bars: 100 µm in in B,D,F,G,J; 15 µm in O. Data are mean±s.e.m. *P<0.05, **P<0.01, ***P<0.001; unpaired Student's *t*-test.

Given the pro-neurogenic effects of the M4 mAChR potentiators, we next investigated whether impairments in hippocampal neurogenesis due to cholinergic neuron loss could be compensated for by enhancing NPC proliferation via stimulation of M4 mAChRs *in vivo*. The basal forebrain cholinergic system comprises the nucleus basalis of Meynert (NBM), the substantia innominata (SI), the horizontal and vertical DBB and the MS, with the MS/DBB providing the primary source of cholinergic innervation to the hippocampus (Auld et al., 2002). A characteristic feature of basal forebrain cholinergic neurons is the expression of high levels of the p75 neurotrophin receptor (Woolf et al., 1989). Intracerebroventricular injections of the immunotoxin p75-Saporin (p75-Sap, an anti-murine p75 monoclonal antibody conjugated to the ribosome-inactivating protein saporin) have previously been used to effectively ablate cholinergic neurons while sparing the neighboring noncholinergic neurons in the basal forebrain (Berger-Sweeney et al., 2001; Hamlin et al., 2013; Moreau et al., 2008). As expected, 2 weeks after injection of p75-Sap or a rabbit-Saporin control (IgG-Sap) into the MS/DBB (Fig. 4A), the majority of MS/DBB cholinergic neurons in mice injected with p75-Sap were lost compared with the number in animals injected with IgG-Sap (Fig. 4B,C). The ablation was selective for the MS/DBB, as the number of cholinergic neurons in the NBM and SI remained unaltered (Fig. 4D,E). The lesion also resulted in a substantial decrease in the cholinergic fiber density in both the molecular and granular cell layers of the hippocampus, as well as in the hilar region (Fig. 4F-H). The overall structural integrity of the dentate gyrus was preserved, with this area found to be similar in IgG-Sap- and p75-Sap-treated mice (Fig. 4I). We also examined neuroinflammation by quantifying the number of Iba1^+^ microglia in the dentate gyrus of IgG-Sap- versus p75-Sap-treated mice and found no difference between groups (Fig. 4J,K). Thus, infusion of p75-Sap into the MS/DBB induces near complete loss of cholinergic neurons and their innervation of the hippocampus, while preserving other basal forebrain cholinergic nuclei.

To evaluate the baseline effects of selective MS/DBB cholinergic lesion on adult hippocampal neurogenesis, we next labeled proliferating cells using BrdU 2 weeks post-lesion, with the mice being sacrificed 3 weeks later (Fig. 4L). We found that the MS/DBB lesion did not significantly alter the number of cells expressing Ki67, an endogenous marker of cell proliferation (Fig. 4M), but resulted in a significant decrease in hippocampal neurogenesis, with fewer BrdU^+^ cells and newly generated neurons (BrdU^+^/DCX^+^) being observed in p75-Sap- compared with IgG-Sap-injected mice (Fig. 4N-P). A significant reduction in the total number of DCX^+^ immature neurons was also observed in these mice (Fig. 4Q). Together, these data indicate that the loss of cholinergic innervation to the hippocampus does not impact the baseline proliferation or neurogenic differentiation of NPCs. Rather, this innervation is important for the survival of any newly generated progenitors and/or neurons, possibly through inputs from MS/DBB cholinergic afferents (Deshpande et al., 2013; Vivar et al., 2012).

**Fig. 5.**
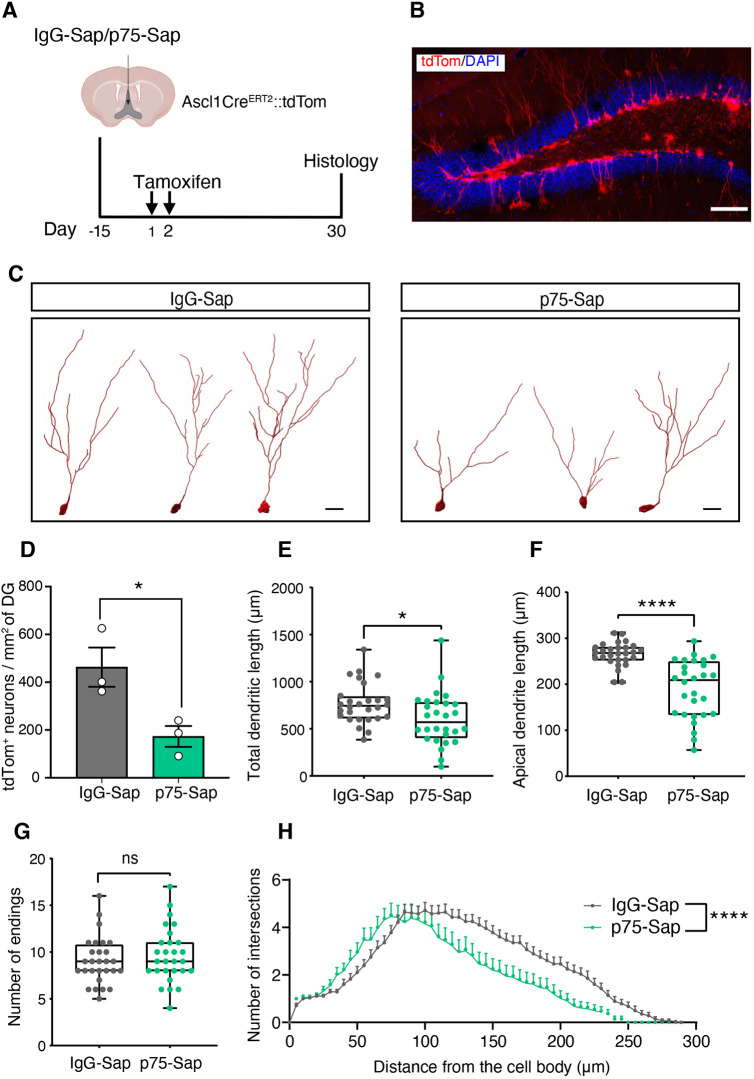
**Loss of MS/DBB cholinergic neurons impairs dendritic development of immature adult-born neurons in the hippocampus.** (A) Experimental timeline showing labeling of adult-born neurons using Ascl1^CreERT2^::tdTom mice. Two weeks after IgG-Sap or p75-Sap infusion in the MS/DBB, mice were injected with tamoxifen for 2 consecutive days to label and lineage trace new neurons. They were then sacrificed 4 weeks later. (B) A confocal image showing adult-born neurons (tdTom^+^, red) and nuclei stained with DAPI (blue) in the hippocampus. Scale bar: 100 µm. (C,D) Representative traces (C) and the number of tdTom^+^ adult-born neurons per mm^2^ of dentate gyrus (D) in IgG-Sap- and p75-Sap-treated mice. (E,F) A significant reduction in the total dendritic length (E) and the length of the apical dendrite (F) was observed in the neurons of the p75-Sap-treated mice. (G) No difference in the number of endings of the immature adult-born neurons was noted between groups. (H) Sholl analysis revealed a significant difference in the dendritic architecture of up to 4-week-old adult-born neurons in p75-Sap-treated compared with control mice. *n*=4 mice per group; 7 neurons per mouse; two-way ANOVA. Data are mean±s.e.m. *P<0.05, ****P<0.0001; unpaired Student's *t*-test (D-F). The boxes represent the interquartile range; whiskers indicate minimum to maximum values. Scale bars: 20 μm. DG, dentate gyrus.

Cholinergic signaling has been shown to play an important role in shaping postnatal neural development, including neuronal maturation and dendritic development (Picciotto et al., 2012). Given that the appropriate maturation and connectivity of newborn neurons is important for their integration into the dentate gyrus circuit, we next investigated whether the loss of MS/DBB cholinergic projections alters the morphological maturation of the surviving newborn hippocampal neurons. To address this, we adopted a genetic strategy involving Ascl1^CreERT2^::tdTom mice to label and lineage trace a cohort of adult-born (tdTom^+^) neurons (Jhaveri et al., 2018; Yang et al., 2015). Two groups of Ascl1^CreERT2^::tdTom mice were set up, with one group receiving a p75-Sap and the other an IgG-Sap injection in the MS/DBB. To characterize the morphology of immature adult-born neurons, these mice were sacrificed 4 weeks after the administration of tamoxifen (Fig. 5A) and the dendrites of individual tdTom^+^ neurons were traced and analyzed (Fig. 5B,C). Consistent with the data reported above, we observed a significant reduction in the number of tdTom^+^ neurons in p75-Sap- compared with IgG-Sap-injected mice (Fig. 5D). In addition, our results revealed a significant reduction in the total dendritic length and the length of the apical (longest) dendrite of tdTom^+^ newborn neurons in the p75-Sap-injected mice compared with the controls (Fig. 5E,F). However, the total number of dendritic endings and intersections were similar between groups (Fig. 5G). Sholl analysis revealed a significant difference in the dendritic arborization between groups, with tdTom^+^ neurons from the p75-Sap group exhibiting branching closer to the cell body than neurons from the IgG-Sap group (Fig. 5H). These data demonstrate that MS/DBB cholinergic innervation is important for the morphological maturation of newborn hippocampal neurons, with its loss leading to impairments in the dendritic development and branching pattern of these cells.

### Stimulating M4 mAChRs does not rescue MS/DBB cholinergic lesion-induced loss of newborn neurons but further exacerbates the impairments in their morphological maturation

**Fig. 6.**
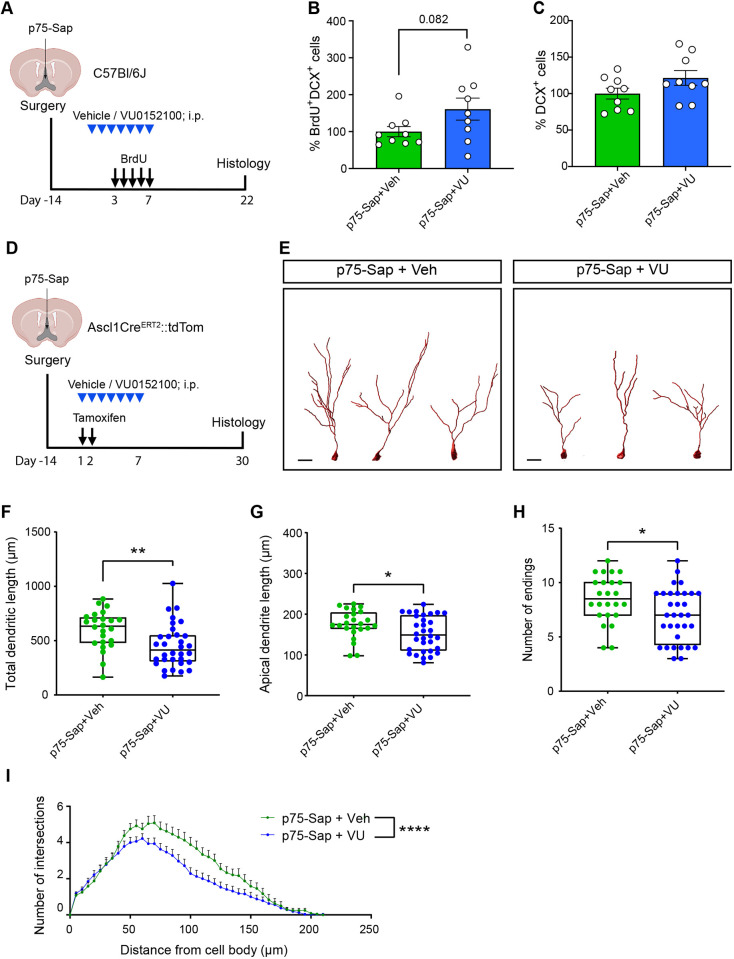
**Effects of M4 mAChR stimulation on adult hippocampal neurogenesis in MS/DBB cholinergic-lesioned mice.** (A) Experimental timeline. 14 days post-p75-Sap infusion, mice were systemically administered either VU0152100 or vehicle daily for a period of 7 days. BrdU was administered daily for 5 consecutive days and mice were sacrificed 3 weeks later. (B,C) Quantification of the number of newborn neurons (B; BrdU^+^DCX^+^) and total immature neurons (C; DCX^+^) in the vehicle- and VU015200-treated mice (unpaired Student's *t*-test). (D) Experimental timeline for assessing the dendritic morphology of newborn neurons following MS/DBB cholinergic lesion and systemic administration of VU0152100 or vehicle using Ascl1^CreERT2^::tdTom mice. (E) Representative tracing of tdTom^+^ adult-born neurons from p75-Sap+Veh- and p75-Sap+VU-treated mice. Scale bars: 20 μm. (F-H) A significant reduction in the total dendritic length (F), length of the apical dendrite (G) and number of endings of the immature neurons (H) was found in p75-Sap+VU- compared with p75-Sap+Veh-treated mice. (I) Sholl analysis also revealed a significant decrease in the dendritic arborization of adult-born neurons in p75-Sap+VU-treated mice (*n*=3 or 4 mice per group, 8 neurons per mouse; two-way ANOVA). Data are mean±s.e.m.: *P<0.05, **P<0.01, ****P<0.0001; unpaired Student's *t*-test (F-H). The boxes represent the interquartile range; whiskers indicate minimum to maximum values.

In order to assess the neurogenic effects of stimulating M4 mAChRs under conditions of MS/DBB cholinergic neuron loss, we next set up two groups of mice in which MS/DBB cholinergic neurons were selectively ablated by administering a single injection of p75-Sap 14 days post-injection, after which the animals were systemically administered either VU0152100 or vehicle daily for a period of 7 days (Fig. 6A). We observed a trend towards an increase in the number of newborn neurons (BrdU^+^DCX^+^) in the p75-Sap+VU0152100-treated mice compared with the p75-Sap+vehicle group (Fig. 6B). However, the total number of immature neurons (DCX^+^) was not significantly different between groups (Fig. 6C), suggesting that pharmacological stimulation of M4 mAChRs could not fully compensate for the MS/DBB cholinergic lesion-induced loss of newborn neurons.

Finally, to examine whether selective M4 mAChR stimulation could restore the appropriate morphological development of newborn neurons after MS/DBB cholinergic lesion, we again used the Ascl1^CreERT2^::tdTom mice (Fig. 6D). After p75-Sap-induced MS/DBB cholinergic neuron lesion, one group of mice received VU0152100 and the other received vehicle for 7 days. To assess the dendritic architecture of up to 4-week-old immature adult-born neurons, the mice were sacrificed 4 weeks after tamoxifen administration. Strikingly, we observed a significant reduction in the total dendritic length and the length of the apical dendrite of the newborn neurons in the VU0152100-treated, p75-Sap-lesioned mice compared with the vehicle-treated p75-Sap-lesioned animals (Fig. 6E-G). The total number of dendritic endings was also significantly reduced (Fig. 6H), with Sholl analysis revealing a further decrease in the number of dendritic intersections in the VU0152100-treated mice compared with the vehicle-treated MS/DBB-lesioned animals (Fig. 6I).

Taken together, these findings suggest that stimulation of M4 mAChRs cannot rescue the MS/DBB cholinergic lesion-induced decrease in the level of hippocampal neurogenesis. In fact, under conditions of greatly impaired cholinergic neurotransmission, M4 mAChR stimulation further exacerbates the impairments in the morphological maturation of adult-born neurons.

## DISCUSSION

The production of new neurons in the dentate gyrus of the adult hippocampus is a multi-stage process that includes the activation and proliferation of NPCs within the neurogenic niche, their specification into neurons, and their functional differentiation and integration within the hippocampal circuit. This study has revealed the contributions of cholinergic signaling during various stages of neurogenesis, including: (1) the expression of M4 mAChRs by NPCs, and the ability of M4 mAChR-selective potentiators to enhance their proliferation, resulting in increased numbers of newborn neurons; (2) the necessity of septal-hippocampal cholinergic neurotransmission for the survival of adult-born neurons; and (3) the ability of cholinergic innervation to sculpt the dendritic architecture of newly generated immature neurons.

Fibers expressing choline acetyltransferase, the enzyme responsible for acetylcholine synthesis, have been observed in close proximity to NPCs in the hippocampal neurogenic niche (Kaneko et al., 2006), suggesting that these NPCs may receive direct inputs from the basal forebrain cholinergic neurons. Previous studies have reported the expression of M1 and M4 mAChRs on Nestin-GFP^+^ cells as well as on a subpopulation of proliferating cells in the dentate gyrus (Itou et al., 2011; Mohapel et al., 2005; Shin et al., 2015). Our study has revealed, for the first time, that M4 mAChRs are selectively expressed and enriched in Nestin-GFP^+^EGFR^+^ compared with Nestin-GFP^+^EGFR^−^ NPCs. Our previous study has shown that the expression of EGFR alone is not sufficient to distinguish between active and quiescent hippocampal NPCs (Jhaveri et al., 2015), and have proposed transactivation of EGFR signaling by a G-protein-coupled receptor (GPCR) family, such as β3-adrenergic receptors (Jhaveri et al., 2010) or KCl acting via L-type calcium channels (Walker et al., 2008), as a mechanism that would ensure the rapid cell cycle entry of activatable (EGFR^+^) precursor cells upon neurogenic stimulation while preserving the non-activatable or dormant (EGFR^−^) precursor pool. Indeed, we found that pharmacological stimulation using M4-selective allosteric modulators (VU10010 and VU0152100) led to enhanced proliferation of these Nestin-GFP^+^EGFR^+^ NPCs *ex vivo*, and resulted in increased production of new neurons in the adult hippocampus *in vivo* in non-lesioned animals. Several cellular mechanisms could potentially explain this M4 mAChR-mediated enhancement in NPC activity, including increased survival and/or proliferation of NPCs, or recruitment of quiescent neural stem cells. Although our data do not allow us to distinguish between these possibilities, the results of the clonal density neurosphere assay indicate a direct effect on Nestin-GFP^+^EGFR^+^ NPCs.

Emerging studies have suggested that the majority of hippocampal NPCs exist in a distinct metabolic state of quiescence (Urbán et al., 2019) and that quiescent hippocampal NPCs are marked by high levels of reactive oxygen species (hiROS) (Adusumilli et al., 2021). Given that hiROS cells make up the majority of neurosphere-forming cells, including those that are responsive to KCl or norepinephrine (Adusumilli et al., 2021), together with our data showing that M4 mAChR stimulation promotes the proliferation of norepinephrine-responsive NPCs, it is tempting to speculate that M4 stimulation may recruit quiescent hippocampal NPCs to proliferate. However, it is also possible that M4 mAChRs are expressed on mitogenically active NPCs and that their stimulation leads to an increased rate of proliferation. Thus, it remains to be determined whether M4 mAChR-expressing Nestin-GFP^+^EGFR^+^ cells represent ‘primed’ or ‘resting’ NPCs or an already ‘activated’ hippocampal NPC population (Harris et al., 2021).

How M4 mAChRs mediate the proliferation of hippocampal NPCs is currently unknown. M4 mAChRs are preferentially coupled to inhibitory G (G_i/o_) proteins, which can link with potassium channels to reduce the resting membrane potential (Ishii and Kurachi, 2006), as well as modulating several signaling pathways linked to altered rates of neurogenesis. For example, the membrane hyperpolarization of ventricular zone NPCs shifts the transcriptional program and neurogenic division mode (Vitali et al., 2018). Conversely, G_i/o_ proteins inhibit adenylate cyclase, leading to a decrease in intracellular cAMP (Langmead et al., 2008), which has been reported to act as a positive regulator of adult hippocampal neurogenesis through increased proliferation (Nakagawa et al., 2002). M4 mAChRs have also been shown to activate the mitogen-activated protein (MAP) kinase pathway, as well as transactivating EGF receptors (Ockenga et al., 2013; Stirnweiss et al., 2006). Overall, our results add to our growing mechanistic understanding of the neurotransmitter-mediated control of NPC activity in the adult hippocampus (Berg et al., 2013; Song et al., 2016).

Our finding that the selective loss of MS/DBB cholinergic neurons in mice results in a significant impairment in adult hippocampal neurogenesis reflects findings previously reported in rats after a global basal forebrain cholinergic lesion (Cooper-Kuhn et al., 2004; Mohapel et al., 2005). This demonstrates that the cholinergic inputs into the hippocampus from the MS/DBB directly regulate neurogenesis and shows that the regulation is not due to an indirect effect of the NBM or SI cholinergic neurons, although this remains a possible additional regulatory mechanism. Whereas our targeted approach resulted in a profound loss of septal-hippocampal innervation and a significant reduction in neurogenesis, a previous study that achieved only partial elimination of the MS/DBB cholinergic neurons reported no effect on baseline hippocampal neurogenesis (Ho et al., 2009). This indicates that a normal to low level of cholinergic innervation is sufficient to support baseline levels of hippocampal neurogenesis. Hence, strategies aimed at promoting the survival of MS/DBB cholinergic neurons, or mimicking the major compromised cholinergic functions, could prove beneficial for the maintenance of ongoing neurogenesis and neurogenesis-dependent functions in various neurodegenerative conditions, including Alzheimer's disease, in which basal forebrain cholinergic neuron loss is one of the key early pathogenic events (Hampel et al., 2019).

We found that the loss of MS/DBB cholinergic innervation in the hippocampus led to a significant decrease in the number of newly generated neurons (BrdU^+^/DCX^+^) without altering the overall proliferation in the dentate gyrus, suggesting reduced survival of newly generated progenitors and/or neurons. Notably, treatment with the M4 mAChR-selective allosteric potentiator could not rescue this phenotype, suggesting involvement of as yet unidentified cholinergic mechanisms that act either directly or indirectly via the neurogenic niche. Our study also revealed that MS/DBB cholinergic neurons are important for the morphological maturation of surviving adult-born neurons, contributing to their structural plasticity. These findings add to the existing literature reporting the contribution of GABA and glutamate in the regulation of the dendritic development of adult-born neurons (Song et al., 2013; Tashiro et al., 2006), highlighting a major role for neurotransmitters in sculpting the morphological and connectivity patterns of these neurons in the hippocampus. In particular, our results from the systemic administration of VU0152100 revealed that the stimulation of M4 mAChRs expressed by NPCs or their progeny exacerbated the reduced dendritic branching induced by loss of cholinergic innervation to the dentate gyrus. Whether M4 mAChRs are also expressed by adult-born neurons during their immature phase is currently not known. However, given the inhibitory effect of M4 mAChRs on the neurotransmission of neurons, it is perhaps not surprising that the maturation and integration of newborn neurons is impaired. Interestingly, both loss of cholinergic function and activation of M2 and M4 mAChRs can suppress the hippocampal theta (or ripple) oscillations associated with hippocampal- and adult neurogenesis-dependent learning and memory (Berdugo-Vega et al., 2020; Kumar et al., 2020; Ma et al., 2020).

Determining how septal cholinergic neurons regulate the morphological development of adult-born neurons in the hippocampus remains challenging, as this function likely involves both direct and indirect mechanisms. Retrograde tracing experiments using pseudo-type rabies virus have demonstrated the presence of significant and stable monosynaptic cholinergic inputs arising from the MS/DBB onto immature adult-born hippocampal neurons, starting as early as 10 days after their birth (Deshpande et al., 2013). Optogenetic stimulation of DBB cholinergic neurons evoked synaptic currents in these immature adult-born neurons, thereby establishing the functional synaptic connections between them (Zhu et al., 2017). An indirect contribution of the basal forebrain cholinergic system to the modulation of adult neurogenesis via the entorhinal cortex has also been proposed (Mesulam et al., 1983). As robust glutamatergic synaptic inputs from the entorhinal cortex onto adult-born neurons are established at a more mature stage, around 3 weeks after their birth (Deshpande et al., 2013; Vivar et al., 2012), the indirect contribution of cholinergic signaling via these afferents may well regulate the later stages of maturation and/or integration of these neurons. Adding another layer of complexity is the finding that cholinergic terminals in the hippocampus co-transmit acetylcholine and GABA (Takács et al., 2018), raising the possibility that the impairments in the morphological development of adult-born neurons observed after MS/DBB cholinergic lesion could also be due to a reduction in GABAergic signaling. In support of this notion, GABAergic synaptic inputs from local parvalbumin-expressing interneurons have previously been shown to promote the survival and morphological development of adult-born neurons in the hippocampus (Song et al., 2013). Although one study reported that the expression of functional M1 mAChRs on immature adult-born neurons is essential for their survival (Zhu et al., 2017), future investigations to examine the full repertoire of cholinergic receptor subtype(s) on immature adult-born neurons and determine their functional roles will be essential for uncovering the contribution of cholinergic signaling to the regulation of the structural plasticity of these cells.

Based on our findings, we propose that the neurogenesis-promoting activity of M4-selective allosteric potentiators could be of therapeutic benefit in conditions where reduced rates of neurogenesis are observed and neurogenesis-dependent functions are impaired, such as during aging and in neurodegenerative conditions. In fact, these M4 mAChRs potentiators have been proposed as promising novel small molecule pharmaceuticals for the treatment of the cognitive and behavioral impairments present in Alzheimer's disease and schizophrenia (Foster and Conn, 2017). There is a growing literature showing an antipsychotic drug-like profile for VU0152100 (Brady et al., 2008; Byun et al., 2014). Furthermore, there is also evidence supporting the beneficial effects of M4 potentiators in preclinical models of associative learning and memory functions (Bubser et al., 2014). However, our data showing exacerbated impairments in the dendritic architecture of adult-born neurons after systemic administration of VU0152100 to MS/DBB-lesioned animals highlights the need to proceed with caution, especially when considering therapeutic strategies for conditions that are associated with the loss of basal forebrain cholinergic neurons.

In summary, we have identified a subpopulation of hippocampal NPCs expressing M4 mAChRs that can be stimulated to promote their proliferation and enhance the production of new neurons. Stimulation of these M4 mAChR-expressing NPCs could not fully rescue the MS/DBB cholinergic lesion-induced decrease in the levels of hippocampal neurogenesis. In fact, our findings uncouple the role of M4 mAChR-selective potentiators in regulating NPC activity from that which supports the morphological maturation of newborn neurons, at least in conditions where there is cholinergic neuron loss. We therefore propose that future studies not only examine the roles of select compounds/genes/signaling in altering the overall level of neurogenesis but also extend their investigation to include an assessment of the structural and functional properties of adult-born neurons to obtain a comprehensive understanding of the stage-specific regulation of neurogenesis and its implications for the modulation of hippocampus-dependent functions.

## MATERIALS AND METHODS

### Animals

Adult (7-9 weeks old) male C57Bl/6J mice (Animal Resources Centre, Australia) were used to examine the effects of selective MS/DBB cholinergic lesion and pharmacological manipulation of acetylcholine receptors on adult hippocampal neurogenesis *in vitro* and *in vivo*. Male and female Nestin-GFP (green fluorescent protein) mice were used for fluorescence-activated cell sorting (FACS), RNA-seq transcriptome analysis and single-molecule fluorescence *in situ* hybridization, and to examine the activation and proliferation of NPCs *in vivo*. These mice express GFP under the control of the Nestin promoter, which allows visualization and purification of resident NPCs (Jhaveri et al., 2015). Tamoxifen-inducible mice under the control of Achaete-scute complex homolog 1 (Ascl1)-Cre^ERT2^ (Ascl1-Cre^ERT2^; Jackson Laboratory, 012882) crossed with CAG floxStop-tdTomato reporter mice (tdTom; obtained from the Jackson Laboratory, 007914) were used to examine the morphological maturation of adult-born neurons. Mice were housed with up to four same-sex littermates in individually ventilated cages. All cages contained bedding and nesting material. Mice were maintained on a 12 h light-dark cycle (lights on at 07:00 h) and supplied with *ad libitum* access to water and food. Experiments were performed in accordance with the Australian Code of Practice for the Care and Use of Animals for Scientific Purposes and were approved by the University of Queensland Animal Ethics Committee (MRI-UQ/TRI/163/17, QBI/566/18 and 2022/AE000296).

### Neurosphere assay

8-9 week-old male C57Bl/6J mice were sacrificed by cervical dislocation, and their brains were removed and collected in ice-cold Hank's essential medium. The hippocampi were microdissected and minced and a single-cell suspension was prepared as previously described (Jhaveri et al., 2015). The resulting pellet was resuspended in 1 ml of complete neurosphere medium containing EGF (20 ng/ml; receptor grade, BD Biosciences) and bFGF (10 ng/ml; recombinant bovine, Roche). The cells were then plated in a 96-well plate in complete neurosphere medium containing DMEM/F-12, EGF and bFGF, with or without muscarine (10 μM, 50 μM), nicotine (10 μM, 50 μM), VU010010 (10 μM), VU0152100 (10 μM) or PD102807, a selective M4 mAChR antagonist (1 μM; Tocris Bioscience). Muscarine was dissolved in 100% DMSO, with the final concentration of DMSO being adjusted to 0.1% for each treatment, including vehicle. Norepinephrine (10 μM) and KCl (15 mM) were used to investigate the effects of VU010010 on subpopulations of quiescent NPCs. The plates were incubated at 37°C and the total number of neurospheres obtained in each treatment group was determined on day 14, with all conditions normalized to the control group for each experimental replicate and plotted as a percentage of the control.

### Fluorescence-activated cell sorting

Brains from 7- to 9-week-old Nestin-GFP mice were used for FACS. A single-cell suspension of hippocampal tissue was prepared as described above. The resulting pellet was resuspended in DMEM/F12 medium and incubated with biotinylated EGF conjugated with Alexa Fluor 647-streptavidin (EGF-647; 2 μg/ml; Life Technologies) for 30 to 40 min at 4°C. The antibody was washed off using excess DMEM/F12 medium. Propidium iodide (1 μg/ml) was then added to label and exclude dead cells. The cell suspension was filtered using a 40 µm filter before sorting. Cells were analyzed and sorted using a FACS Aria sorter (Becton Dickinson). The positive gates were set relative to the basal fluorescence levels obtained from wild-type littermates and single-fluorescence controls. Nestin-GFP^+^ cells co-expressing EGF receptor (Nes-GFP^+^EGFR^+^), which represent a near pure population of NPCs (Jhaveri et al., 2015), were sorted and collected in a tube containing neurosphere medium. Cells were plated in 96-well plates at a clonal density (<1 cell per well) in complete neurosphere medium. A total of 24 wells were plated per condition (control, muscarine and muscarine+VU10010). Neurospheres were counted on day 14 and conditions were normalized to the control group.

### Single-molecule fluorescence *in situ* hybridization

Nestin-GFP mice were perfused transcardially with PBS and 4% PFA. Brains were dissected and fixed in PFA for 24 h. For cryopreservation, fixed brains were first immersed in 15% sucrose and then in 30% sucrose, after which they were embedded in optimal cutting temperature (OCT) compound and stored at −80°C. The brains were sectioned using a cryostat (CryoStar NX70) at 16 µm, collected on UberFrost slides (InstrumeC), air dried at −30°C overnight and then stored at −80°C. To detect Chrm4 mRNA, we used a validated mouse Chrm4 RNAscope probe (Advanced Cell Diagnostics). Fluorescence *in situ* hybridization was performed according to the manufacturer's specifications (RNAscope Fluorescent Multiplex Reagent Kit part 2, Advanced Cell Diagnostics) with the following modifications: incubation with 10% neutral buffered formalin for 5 min at 4°C instead of 15 min at room temperature and incubation with target retrieval solution for 10 min instead of 5 min. For each fluorescence *in situ* hybridization experiment, we included the RNAscope 3-plex positive control mouse probe mix (ACD 320881) and 3-plex negative control probe mix. To visualize Nestin-GFP^+^ cells after fluorescence *in situ* hybridization, sections were blocked in 10% normal donkey serum in PBT (0.2% Triton X-100 in PBS) for 1 h at room temperature, followed by 2 h incubation with AlexaFluor488 anti-GFP antibody (Biolegend) and then 3×5 min washes in PBT.

RNA fluorescence *in situ* hybridization was visualized and imaged using an Olympus UPLXAPO 20x/0.8 NA air objective on a spinning disk confocal microscope (SpinSR10) built around an Olympus IX3 body equipped with two ORCA-Fusion BT sCMOS cameras (Hamamatsu Photonics) and controlled by Olympus cellSens software. *Z*-stack images were acquired using the SoRa super- resolution disk at 3.2× magnification. All image processing and analysis post-acquisition were performed using Fiji for Windows (ImageJ 1.52d). We established a threshold above the background to define Chrm4^+^ cells and quantified the proportion of Nestin-GFP^+^ cells expressing Chrm4 transcripts in two hippocampal sections per animal.

### Labeling newborn cells

To genetically label NPCs and trace their progeny, Ascl1-Cre^ERT2^::tdTom mice were administered tamoxifen (Sigma-Aldrich) i.p. at 150 mg/kg on 2 consecutive days. The tamoxifen was dissolved in corn oil (Sigma-Aldrich) with 10% ethanol at 50 mg/ml concentration. To label proliferating cells *in vivo*, 5-bromo-2-deoxyuridine (BrdU; 100 mg/kg; i.p., Sigma-Aldrich) was injected daily for 5 consecutive days.

### Intrahippocampal and systemic stimulation of M4 mAChRs

To selectively stimulate hippocampal M4 mAChRs, 10 μM VU10010 (Tocris), a selective allosteric potentiator (Shirey et al., 2008), was infused into the hippocampus using a single cannula attached to a 7 day, 0.5 μl/h osmotic pump (Alzet Model 1007D). Vehicle (0.1% DMSO, 0.2% bovine serum albumin in saline) was infused in the control group of mice. The coordinates from Bregma were: anterior/posterior, −2.0 mm; medial/lateral, −1.3 mm; and dorsal/ventral, −2.1 mm. The osmotic pump was subcutaneously implanted caudal to the scapula. The mice were perfused 15 days after the end of the infusion period and their brains were harvested for immunohistochemical analysis. To evaluate the effects of the blood-brain barrier-permeable, potent and selective Chrm4 allosteric modulator VU0152100 (Brady et al., 2008) on NPCs, Nestin-GFP mice were treated with VU0152100 (0.5 mg/kg, Sigma-Aldrich) or vehicle via daily i.p. injections for 5 consecutive days and were perfused on day 6. To examine its effects on adult hippocampal neurogenesis, C57Bl/6J mice were treated with VU0152100 or vehicle via daily i.p. injections for 7 consecutive days. Mice were perfused 15 days after the end of the treatment and their brains were collected for histology. The VU0152100 was dissolved in 40% PEG800, 5% Tween20 and 10% DMSO in saline.

### Medial septum cholinergic neuron lesion

7- to 9-week-old C57Bl/6J and Ascl1-Cre^ERT2^::tdTom mice were anesthetized with an intraperitoneal (i.p.) injection of ketamine/xylazine (50 mg/kg and 8 mg/kg body weight, respectively). Infusion of murine-p75 neurotrophin receptor (p75NTR)-Saporin (hereafter abbreviated as p75-Sap; 0.4 µg/µl; Advanced Targeting System) or control rabbit-IgG-Saporin (IgG-Sap; 0.4 µg/µl) was performed using a 30G needle attached to a 5 µl Hamilton syringe. The stereotaxic coordinates from Bregma for MS injections were: anterior/posterior, −0.9 mm; medial/lateral, 0 mm; and dorsal/ventral, −4.7 mm. The toxin was infused at a rate of 0.4 μl/min (the total volume injected was 1.5 μl), with the needle left in place for 10 min to allow for diffusion. Animals were administered Temgesic (buprenorphine; 0.l mg/kg; subcutaneous injection; Temvet, Ilium) for postoperative pain relief.

### Tissue collection and immunohistochemistry

Mice were perfused transcardially using ice-cold 4% paraformaldehyde (PFA). Their brains were removed and post-fixed in 4% PFA for 2 h, then left in 30% sucrose for up to 48 h, after which 40 μm sections were cut using a freezing microtome. The sections were washed in 0.1 M PBS three times and stored at 4°C with 0.01% sodium azide. Every sixth section was used for immunohistochemical analysis. For BrdU immunolabeling, the sections were rinsed in 0.1 M PBS and incubated in 1 M HCl at 45°C for 20 min. Sections were rinsed in boric acid (pH 6) for 5-10 min, followed by a wash in 0.1 M PBS. For BrdU and Nestin-GFP co-labeling, an additional antigen retrieval step was performed using pre-heated sodium citrate buffer (10 mM) and sections were incubated at 70°C for 30 min. They were then placed into a blocking solution containing 0.1% Triton X-100 in PBS (0.1% PBST) and 5% normal goat serum for 1.5 h at room temperature. The sections were subsequently incubated overnight in solution containing primary antibodies, including mouse anti-BrdU antibody (1:500; Roche, 11170376001), rat anti-BrdU antibody (1:500; BioRad, OBT0030), chicken anti-GFP (1:1000; Invitrogen, A10262), rabbit anti-p75 neurotrophic receptor (p75NTR; 1:1000; Millipore, 07-476), rabbit anti-doublecortin (DCX; 1:500; Cell Signaling Technology, 4604S), rabbit anti-Ki67 (1:500; Novocastra, NCL-Ki67p), goat anti-tdTomato (1:500; SICGEN, AB8181-200) and rabbit anti-Iba1(1:1000; Fujifilm, 019-19741). The sections were then washed in 0.1% PBST and incubated in the appropriate species-specific secondary antibody for 2 h at room temperature (goat anti-rabbit Alexa 488, Invitrogen, A-11008, 1:2000; goat anti-mouse Alexa 647, Invitrogen, A-21236, 1:2000; donkey anti-goat Alexa 568, Jackson ImmunoResearch, 705586147, 1:2000; goat anti-chicken Alexa 488, Invitrogen, A-11039, 1:2000 and goat anti-rat Alexa 568, Invitrogen, A-11077, 1:2000) and 4′,6-diamidino-2-phenylindole (DAPI, 1:5000, Life Technologies). After several washes, the sections were mounted using fluoromount (DakoCytomation) and viewed on a Zeiss-Axio Imager microscope or Diskovery spinning disk confocal microscope (Andor Technology) built around a Nikon Ti-E body (Nikon Corporation) and equipped with two Zyla 4.2 sCMOS cameras (Andor Technology) and controlled by Nikon NIS software.

### Microscopy and image analysis

To visualize p75NTR^+^ cells in the basal forebrain nuclei, images were acquired using an upright fluorescence slide scanner (Metafer VSlide Scanner by MetaSystems using Zeiss Axio Imager Z2) with a 20× air objective. p75NTR^+^ cells in the MS/DBB, NBM and SI were quantified using Imaris software (Version 9.3). For experiments evaluating neurogenesis and adult-born neuron morphology, only animals with more than a 50% loss of p75NTR^+^ cells in the MS/DBB following p75-Sap injection were included in the study. Fluorescence immunolabeling in tissue was visualized and imaged using a Nikon Plan Apochromat 20×/0.75 NA air objective and a Plan Apo Lambda 60x/1.4 NA oil-immersion objective on a Diskovery spinning disk confocal microscope. Representative images of BrdU, DCX, Nestin-GFP, Iba1 or tdTomato staining in the dentate gyrus were taken using a 20× objective at 0.5-2 μm intervals. For morphological analysis, three-dimensional images of dendrites were obtained from *z*-stacks of confocal images taken at 0.5 μm intervals using a 60× oil immersion objective. To accurately reconstruct the dendritic morphology, we selected tdTom^+^ newborn neurons that were entirely contained within the tissue section. Morphological analysis was performed using the Imaris software.

### Stereology and quantification

An upright stereology microscope (MicroBrightField Bioscience) built around a Zeiss Axio Imager.Z2 upright microscope body with an ORCA-R2 digital charge-coupled device camera (Hamamatsu Photonics) for fluorescence imaging and a 40× objective lens (Zeiss EC Plan-Neofluar 40×/0.75 NA air objective) was used to perform cell counts. Stereo Investigator software (version 2017.03.3; MicroBrightField Bioscience) was used to quantify cells expressing single markers (p75NTR, BrdU, DCX, Ki67, Iba1 or tdTom) as well as those showing colocalization (BrdU/DCX or Nestin-GFP/BrdU). A minimum of six sections per brain was used and cell quantification was performed without any knowledge of the experimental conditions. Cell numbers in the dentate gyrus were divided by the length of the subgranular zone and the data were expressed as ‘percentage change’ relative to the control.

### Statistical analysis

Statistical analyses were performed using GraphPad Prism 9 (GraphPad). Normally distributed data were analyzed using a two-tailed Student's *t*-test when comparing two groups or a one-way or two-way ANOVA followed by Bonferroni's multiple comparison post-hoc test when comparing more than two groups. For non-normally distributed data, a Log-rank test was used. Differences with *P*<0.05 were considered statistically significant. All data are presented as the mean±s.e.m.
