# Supplementary material for: Stimulation of the muscarinic receptor M4 regulates neural precursor cell proliferation and promotes adult hippocampal neurogenesis
Source: Development. 2024 Jan 2;151(1):dev201835. doi: 10.1242/dev.201835 (PMC10820734; doi:10.1242/dev.201835)
Supplement: Supplementary file 1 [file develop-151-201835_review_history.pdf]

Stimulation of the muscarinic receptor M4 regulates neural precursor cell proliferation and promotes adult hippocampal neurogenesis

Lidia I. Madrid, Katelyn Hafey, Saurabh Bandhavkar, Gabriela O. Bodea, Javier Jimenez-Martin, Michael Milne, Tara L. Walker, Geoffrey J. Faulkner, Elizabeth J. Coulson and Dhanisha J. Jhaveri
DOI: 10.1242/dev.201835

Editor: Francois Guillemot

Review timeline

Original submission:	3 April 2023
Editorial decision:	12 May 2023
First revision received:	4 October 2023
Editorial decision:	30 October 2023
Second revision received:	26 November 2023
Accepted:	1 December 2023

Original submission

First decision letter

MS ID#: DEVELOP/2023/201835

MS TITLE: Stimulation of the muscarinic receptor M4 activates quiescent neural precursor cells and partially ameliorates medial septum cholinergic lesion-induced impairments in adult hippocampal neurogenesis

AUTHORS: Lidia Madrid, Saurabh Bandhavkar, Katelyn Hafey, Javier Jimenez-Martin, Michael Milne, Elizabeth J Coulson, and Dhanisha J Jhaveri

I have now received the reports of three referees on your manuscript and I have reached a decision. The referees' comments are appended below, or you can access them online: please go to BenchPress and click on the 'Manuscripts with Decisions' queue in the Author Area.

As you will see, all the referees express great interest in your work, but they also have significant criticisms and recommend a substantial revision of your manuscript before we can consider publication. In particular, they comment on the lack of evidence that the pharmacological manipulation of M4 receptors acts directly on neural stem cells rather than indirectly via niche cells, and suggests ways to address this experimentally. They also recommend that you investigate the expression of M4 receptors more thoroughly. If you are able to revise the manuscript along the lines suggested by the referees, which may involve further experiments, I will be happy receive a revised version of the manuscript. Your revised paper will be re-reviewed by one or more of the original referees, and acceptance of your manuscript will depend on your addressing satisfactorily the reviewers' major concerns. Please also note that Development will normally permit only one round of major revision. If it would be helpful, you are welcome to contact us to discuss your revision in greater detail. Please send us a point-by-point response indicating your plans for addressing the referee’s comments, and we will look over this and provide further guidance.

Please attend to all of the reviewers' comments and ensure that you clearly highlight all changes made in the revised manuscript. Please avoid using 'Tracked changes' in Word files as these are lost

© 2024. Published by The Company of Biologists under the terms of the Creative Commons Attribution License (https://creativecommons.org/licenses/by/4.0/).

in PDF conversion. I should be grateful if you would also provide a point-by-point response detailing how you have dealt with the points raised by the reviewers in the 'Response to Reviewers' box. If you do not agree with any of their criticisms or suggestions please explain clearly why this is so.

Reviewer 1

Advance summary and potential significance to field

In their manuscript «Stimulation of the muscarinic receptor M4 activates quiescent neural precursor cells and partially ameliorates medial septum cholinergic lesion-induced impairments in adult hippocampal neurogenesis" Madrid and colleagues used a pharmacological approach to describe a role for M4 muscarinic receptors to regulate the activity of adult hippocampal neural stem cells (NSCs) and subsequent steps of neuronal differentiation. The data shown will add to the current knowledge (and quite extensive literature, as discussed by the authors) on cholinergic regulation of hippocampal NSCs. The data shown are convincing - albeit in some aspects not entirely novel; see for example Mohapel et al. 2005 Neurobiol Aging. Thus, the advance provided is somewhat limited; but will still be of interest to the field.

Comments for the author

The authors should address several points to strengthen their manuscript.

- The expression of M4 muscarinic receptors in quiescent NSCs is only indirectly shown (e.g., Fig 1H). The authors should i) add expression data using established approaches to induce quiescence in adult NSCs (e.g., using BMP signaling as described before, for example by Gage, Guillemot groups) and ii) they should mine existing expression data (for example single cell RNA-seq data generated by the Song, Jessberger, Linarsson, Bonaguidi groups) to show expression of M4 in adult quiescent (!) NSCs in vivo. Alternatively, they may use in situ hybridization approaches to show expression of M4 across the neurogenic lineage in the adult DG

- We understand that the data are based on a pharmacological approach (that is certainly fine, even though a genetic approach would have been desirable). However, given the expression of muscarinergic receptors in a few other cell types (e.g., classical astrocytes) within the hippocampus it will be interesting to use again in vitro models of quiescence (see above) to directly probe the effects of M4 stimulation/inhibition in such a "controlled" in vitro system.

- The authors nicely present results suggesting stage-dependent effects upon manipulation of M4 in the developmental course of neurogenesis. However, basically all the analyses presented are based on rather short chases after – for example – BrdU labelling (<30 days). However, to assess true rates of neurogenesis (i.e., stably integrated newborn granule cells) the authors should either add later timepoints after BrdU injection (for selected key experiments with a chase > approx. 30 days) - or analyze not only shape but also numbers of cells generated (e.g., using existing data/tissues as shown in Figure 4). In its current state it remains somewhat unclear if the pharmacological manipulation has "lasting" effects on neurogenesis (e.g., what is the net effect on the number of neurons that are truly integrated).

Reviewer 2

Advance summary and potential significance to field

The authors build on previous results addressing the importance of the cholinergic input to the dentate gyrus in the regulation of adult hippocampal neurogenesis.
The focus is on muscarinic receptor 4 (M4) and in vitro and in vivo approaches are employed to make the case that this receptor drives direct effects of cholinergic input on NPCs and newborn neurons of the hipocampal neurogenic niche.

Comments for the author

© 2024. Published by The Company of Biologists under the terms of the Creative Commons Attribution License (https://creativecommons.org/licenses/by/4.0/).

The focus is on muscarinic receptor 4 (M4) and in vitro and in vivo approaches are employed to make the case that this receptor drives direct effects of cholinergic input on NPCs and newborn neurons of the hipocampal neurogenic niche. The variety of approaches and experimental setups is to be appreciated.
However, the manuscript lacks a bit of focus and a variety of scattered experiments in vivo and in vitro could be traded for a more thorough and deeper analysis.

Importantly selective loss-of-function of the muscarinic receptors in specific cell types (NSCS, NPCs, immature neurons) are missing. Even if the expression of muscarinic receptors has been previously shown and the notion is reinforced herein by in vitro work, the results presented here cannot negate the possibility that the observed effects could be mediated indirectly by many other cell types in the niche. I understand that doing these experiments represents a major effort might be out of the scope of this work. At least, it should be crystal-clear what is known about the expression of the muscarinic receptors in the cell types of interest in vivo, from previous literature and available sequencing data if direct expression by unambiguous immunostaining or similar is not available.

As the authors write in the discussion: "Although a rapid rise in intracellular calcium levels in NPCs has been reported following the application of acetylcholine to hippocampal slices (Itou et al., 2011), whether acetylcholine directly regulates the activity of distinct subpopulations of hippocampal NPCs (Jhaveri et al., 2015) is currently unknown." Unfortunately, the current manuscript does not answer this question either.

This idea has to be clear through the text. As an example, statements such as "… by direct innervation and through M4 mAChRs" must be toned down. The word "directly" is used too much. And the same applies to "selective" as in "Selective stimulation of the M4 mAChR enhances adult hippocampal neurogenesis in Vivo" because is broad stimulation of all the cells with the receptor and it is important that it is not confused with a selective activation of NSPCs.
Another important caveat is that it is not clear whether the focus are the NSPCs (as shown by the title) of the newborn/immature neurons. In the ablation and ablation + stimulation experiments overall cell proliferation but no NSPCs activation was assessed in contrast to the previous experiments. The experiments all must be systematic and consistent, especially if claims about proliferation vs. survival/differentiation are to be made.

In the in vitro experiments, it is not clear what populations are the authors referring to with Nestin-GFP+EGFR− cells vs Nestin- GFP+EGFR+ cells. What would be their in vivo counterparts? A more factual characterization of these populations in vitro is required given the results regarding CHRM4 expresion and to make a solid case.

The case is similar for the KCL vs. norepinephrine. What is the addition of this experiment? What in vivo cell populations are these cells representing?

in the presence of, suggesting that stimulation of M4 mAChRs activates the same subpopulation of hippocampal NPCs as that which is responsive to norepinephrine."

Related to this, the term "quiescent" should be used more carefully, no analysis of cell re-entry of first-time activation was done, so just avoid it when unnecessary.

It would be useful to at least check cell proliferation with any marker to confirm that cholinergic stimulation increases the number of neurospheres by promoting cell division.
Also, in Fig. 1 it would be better to plot the control(s) with their SEM too and run the correspondent statistical tests for several groups.

In the ablation experiments it has to be explored and ruled out that neuronal death and degeneration is not affecting the neurogenic niche rather than just the absence of muscarinic innervation. Checking local neuroinflammation in the DG would help. The specificity of the injection and subsequent ablation is convincing, but a better control, and more informative for the manuscript, would be to ablate cells in nearby nuclei (NBM and SI).

© 2024. Published by The Company of Biologists under the terms of the Creative Commons Attribution License (https://creativecommons.org/licenses/by/4.0/).

Figure 2B; C, E and F would be more informative showing total numbers rather than percentage. It can lead to confusion (BrdU+DCX+ percentage of BrdU+ or of DCX+…?). Also in Fig. 3K-N…

All the “data not shown” data should be shown or not mentioned at all.

The title should reflect that what the papers shows is a variety of effects direct or indirect on several processes of the neurogenic cascade.

Reviewer 3

Advance summary and potential significance to field

Adult hippocampus neurogenesis has been established to be a key mechanism in hippocampal function. This process is impaired in aging and neurodegenerative disorders. Creating a better understanding of mechanisms regulating hippocampal neurogenesis remains a high priority area. While overall signaling is known to affect hippocampal neurogenesis, the contribution of MS and DBB neurons, two major populations of cholinergic neurons in the BF, and the identity of the cholinergic receptor subtypes remain to be established. This manuscript describes studies demonstrating that proliferation of quiescent, hippocampal NPCs is affected by MS and DBB neurons and that these effects are mediated, at least in part, through activation of M4 mAChRs. An unexpected finding was that administration of an M4-selective allosteric potentiator, in the context of cholinergic dysfunction, impaired dendritic morphological maturation of newly born hippocampal neurons. This study makes a key set of contributions to our understanding of basal forebrain cholinergic neuron regulation of fundamental areas of hippocampal neurogenesis and will have a broad contributor impact for this field. The paper is well written and the methods are clearly presented. The statistical approaches seem to be appropriate.

Comments for the author

In the Introduction it would be helpful to clarify what is known about M4 receptor expression by NPCs or whether this study is the first to demonstrate that NPCs expression the M4 subtype.
In the first paragraph of the Results, it would be helpful to define “activity” of NPCs as meaning proliferation. In this paragraph, the point is made that it is not known if acetylcholine directly affects proliferation of NPCs, yet in the Introduction the observation is cited that stimulation of M1 receptors promote NPC proliferation; this is a bit confusing. Perhaps the current state of knowledge on effects of targeting cholinergic receptors on NPCs could be better clarified.
Treatment of NPCs with 50 uM muscarine. Is this a traditional concentration? Is it physiologically relevant and/or in line with its Kd? This seems like a high concentration.
Page 6, second paragraph, NPCs were plated at “clonal density”. It would be helpful to know what clonal density is and the rational for its use.
The combination of the in vitro and in vivo studies is a strong point. The finding that loss of cholinergic innervation to the hippocampus affects survival of newly generated progenitors or neurons more than baseline proliferation or neurogenic differentiation is a key finding.
Page 11, Discussion, top paragraph. Is the present study the first to show directly using NPCs, that cholinergic receptor stimulation, of any form, can promote NPC proliferation or neurogenesis?
A valuable part of the Discussion with broad implication is that strategies promoting survival or functions of MS/DBB cholinergic neurons could serve to maintain ongoing neurogenesis in AD and other pathological settings. Another very valuable point is that suggestion that any targeting of M4 related mechanisms must examine structural and functional aspects of NPCs in addition to traditional measures of proliferation or neurogenesis.
Are any cognitive effects in the various mouse models of M4 potentiators known?
Figures. Overall, the figures are well presented. For Figure 1C, it would be helpful to better describe the measures in the Y axis.

First revision

© 2024. Published by The Company of Biologists under the terms of the Creative Commons Attribution License (https://creativecommons.org/licenses/by/4.0/).

Author response to reviewers' comments

Response to Reviewers – MS ID#: DEVELOP/2023/201835

We thank the reviewers for their constructive comments and questions, which we have addressed below.

Reviewer 1 Advance Summary and Potential Significance to Field:
In their manuscript "Stimulation of the muscarinic receptor M4 activates quiescent neural precursor cells and partially ameliorates medial septum cholinergic lesion-induced impairments in adult hippocampal neurogenesis" Madrid and colleagues used a pharmacological approach to describe a role for M4 muscarinic receptors to regulate the activity of adult hippocampal neural stem cells (NSCs) and subsequent steps of neuronal differentiation. The data shown will add to the current knowledge (and quite extensive literature, as discussed by the authors) on cholinergic regulation of hippocampal NSCs. The data shown are convincing - albeit in some aspects not entirely novel; see for example Mohapel et al. 2005 Neurobiol Aging. Thus, the advance provided is somewhat limited; but will still be of interest to the field.

Response to Reviewer 1

1. The expression of M4 muscarinic receptors in quiescent NSCs is only indirectly shown (e.g., Fig 1H). The authors should i) add expression data using established approaches to induce quiescence in adult NSCs (e.g., using BMP signaling as described before, for example by Gage, Guillemot groups) and ii) they should mine existing expression data (for example single cell RNA-seq data generated by the Song, Jessberger, Linarsson, Bonaguidi groups) to show expression of M4 in adult quiescent (!) NSCs in vivo. Alternatively, they may use in situ hybridization approaches to show expression of M4 across the neurogenic lineage in the adult DG

In this study, we believe that we do provide direct evidence for the expression of M4 muscarinic receptors in quiescent neural precursor cells (NPCs). This is supported by our data showing enrichment and expression of *CHRM4* in Nestin-GFP^{+}/EGFR^{+} cells (Fig. 1C), which is a near-pure population of hippocampal NPCs comprising two subpopulations of quiescent NPCs, one which is activated by norepinephrine and one that is responsive to KCl (Jhaveri et al., *J. Neuroscience*, 2015).

However, to obtain further evidence confirming the expression of M4 muscarinic receptors in the hippocampal NPCs, we conducted additional experiments using qPCR and RNAScope in situ hybridisation approaches. Total RNA was extracted from independent samples (not those used for RNA-seq) of purified (using flow cytometry) populations of Nes-GFP^{+}EGFR^{+} and Nes-GFP^{+} EGFR^{-} cells and used to conduct qPCR analysis. A significant enrichment of Chrm4 mRNA was observed in the Nes-GFP^{+}EGFR^{+} compared to Nes-GFP^{+}EGFR^{-} cells.

© 2024. Published by The Company of Biologists under the terms of the Creative Commons Attribution License (https://creativecommons.org/licenses/by/4.0/).

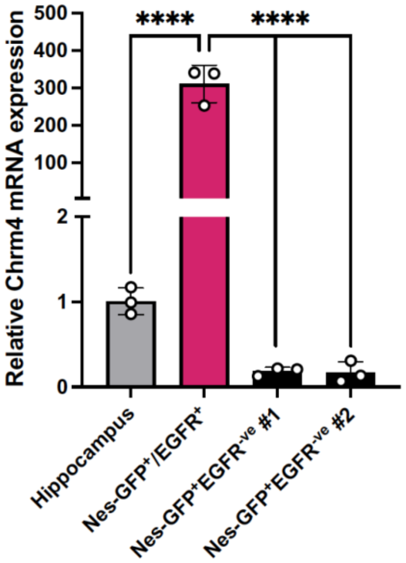

Chrm4 mRNA abundance measured by qPCR in purified Nes-GFP^{+}EGFR^{+} and Nes-GFP^{+} EGFR^{-ve} cells as well as bulk hippocampus. Note the significant enrichment of *CHRM4* transcripts in the Nes-GFP^{+}EGFR^{+} compared to Nes-GFP^{+}EGFR^{-ve} cells. Data are relative to GAPDH and presented as mean ± SEM. ****P<0.0001, one-way ANOVA with Tukey multiple corrections, N=3 technical replicates. # marks biological replicates.

In addition, we performed single-molecule fluorescence *in situ* hybridization (FISH) using a validated mouse Chrm4 RNAscope probe (see revised Materials and methods). Our FISH data confirms the presence of Chrm4 transcripts in a subpopulation of Nestin-GFP^{+} cells, including those exhibiting radial glial like (RGL) morphology (see below). These additional data are included in the revised manuscript (Fig. 1D) and we have dedicated this figure to show the expression of Chrm4 in the hippocampal NPCs. Data demonstrating the effects of stimulating M4 muscarinic acetylcholine receptors (mAChRs) on hippocampal NPCs are now presented in the new Figure 2.

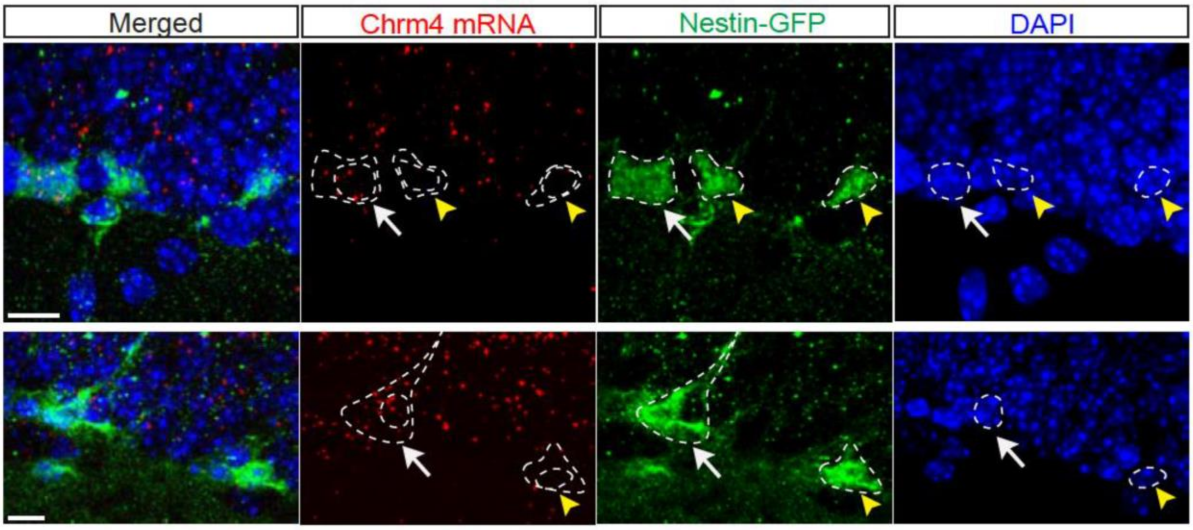

Representative confocal images showing Chrm4 transcripts (red) detected by RNA FISH, Nestin-GFP immunohistochemistry (green) and DAPI staining for nuclei (blue) in the dentate gyrus. Dashed lines in images demarcate nuclear and cellular boundaries defined for DAPI and Nestin-GFP. Note the Chrm4 transcripts in a subpopulation of Nestin-GFP cells (arrows) including the Nestin-GFP^{+} cells showing RGL morphology. Arrowheads point to Nestin-GFP^{+} cells having no Chrm4 transcripts. Scale bars: 10 µm.

© 2024. Published by The Company of Biologists under the terms of the Creative Commons Attribution License (https://creativecommons.org/licenses/by/4.0/).

Collectively, our RNA-seq, qPCR and RNA FISH data provide strong evidence supporting the expression of Chrm4 transcripts in a subpopulation of hippocampal NPCs.

2. We understand that the data are based on a pharmacological approach (that is certainly fine, even though a genetic approach would have been desirable). However, given the expression of muscarinergic receptors in a few other cell types (e.g., classical astrocytes) within the hippocampus it will be interesting to use again in vitro models of quiescence (see above) to directly probe the effects of M4 stimulation/inhibition in such a "controlled" in vitro system.

We believe that the data presented in this study using the neurosphere assay on NPCs isolated from the hippocampus directly address this point. Specifically, purification of Nestin-GFP$^+$/ EGFR$^+$ NPCs from the adult hippocampus using flow cytometry and subsequent culturing of these cells at a clonal density (<1 cell per well) enabled us to probe the direct effects of stimulating M4 muscarinic acetylcholine receptors (mAChRs) directly on hippocampal NPCs (in the absence of any niche cells). Our data showing that treatment with the M4 mAChR-specific allosteric modulator VU10010 in the presence of muscarine led to a significant increase in the number of neurospheres in the clonal density assay compared to the control (Fig. 2D) provide evidence supporting a direct effect.

Nonetheless, based on the reviewer's suggestion, we conducted an additional experiment using an *in vitro* model of quiescence. For this, we used an NPC line that was previously generated from the dentate gyri of adult male C57BL/6JRj mice (Ichwan et al., *Stem Cell Reports*, 2021) and maintained as an adherent monolayer culture as described previously (Ichwan et al., *Stem Cell Reports,* 2021; Adusumilli et al., *Cell Stem Cell*, 2021). Vials of adherent NPCs from passage 7 were removed from liquid nitrogen storage, quickly defrosted at 37°C and the cells diluted to 10 ml with growth medium (neural basal medium containing 2% B-27® supplement (50X), penicillin/streptomycin (100 U/ml), 1% GlutaMAX™, 20 ng/ml epidermal growth factor (EGF) and 20 ng/ml fibroblast growth factor 2 (FGF-2). The cells were centrifuged at 300 x g for 5 min and the pellet was resuspended in 5 ml of growth medium, seeded into a poly-D-lysine (PDL)/ laminin-coated T25 flask and incubated at 37°C with 5% CO_2 for 3 days. Approximately 24 h after plating, once the cells had adhered to the coated surface, the growth medium was exchanged. When the cells had reached 80% confluency, they were passaged by removing the growth medium, washing once with PBS then treating with Accutase for 3 min at 37°C to break the cell contacts. The cells were washed with 10 ml neural basal medium, resuspended in growth medium and plated at a density of 20,000 cells per well onto PDL/laminin-coated coverslips in 24-well plates. To induce quiescence, cells were treated with 20 ng/ml BMP4 for 3 days. Proliferation was assessed using a pulse of BrdU for 2 h prior to fixation. However, before probing the effects of M4 mAChR stimulation on quiescent NPCs using adherent NPC cultures, we conducted qPCR analysis to assess the expression of *CHRM4* in these cells. Interestingly, as shown below, we found that these cells had no or very negligible expression of *CHRM4,* thereby making the stimulation experiment untenable.

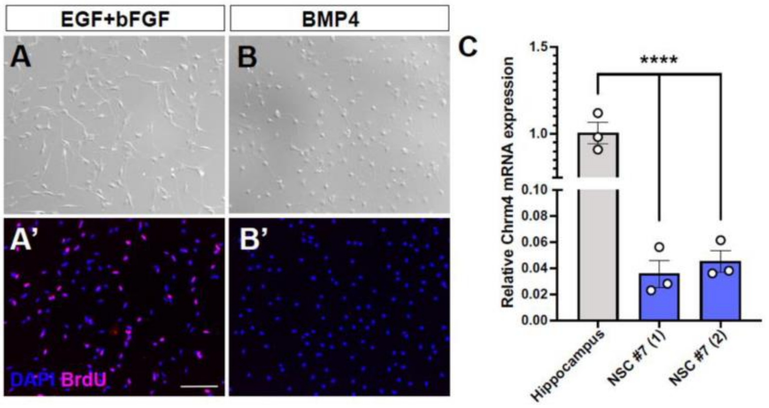

(A, B) Example brightfield images of adherent NPCs in the presence of EGF+bFGF (A) and BMP4 (B). BrdU (pink) incorporation seen in NPCs cultured in EGF+bFGF (A') but not in the BMP4-treated cultures (B'). Nuclei are counterstained using DAPI (blue). (C) Chrm4 mRNA abundance measured by

© 2024. Published by The Company of Biologists under the terms of the Creative Commons Attribution License (https://creativecommons.org/licenses/by/4.0/).

qPCR. Note negligible expression of *CHRM4* in adherent NPC cultures passage #7 (1) and (2) compared to the hippocampus. Scale bar:100 µm. Data are relative to GAPDH and presented as mean ± SEM. ****P<0.0001, one-way ANOVA with Tukey multiple corrections, N=3 technical replicates.

Thus, although the BMP4-induced model provides a "controlled" *in vitro* system to interrogate mechanisms regulating quiescence, based on the data presented above and in the manuscript, we believe that these passaged hippocampal NPCs do not faithfully reflect those present *in vivo,* at least with regards to the expression of Chrm4.

3. The authors nicely present results suggesting stage-dependent effects upon manipulation of M4 in the developmental course of neurogenesis. However, basically all the analyses presented are based on rather short chases after - for example - BrdU labelling (<30 days). However, to assess true rates of neurogenesis (i.e., stably integrated newborn granule cells) the authors should either add later timepoints after BrdU injection (for selected key experiments with a chase > approx. 30 days) - or analyze not only shape but also numbers of cells generated (e.g., using existing data/tissues as shown in Figure 4). In its current state it remains somewhat unclear if the pharmacological manipulation has "lasting" effects on neurogenesis (e.g., what is the net effect on the number of neurons that are truly integrated).

Using two independent pharmacological agents, we have shown that stimulation of M4 mAChRs leads to an increase in the production of new neurons at 21 days following treatment. Given that the majority of cell death during adult hippocampal neurogenesis occurs either immediately (a large fraction of cells undergoes death around 1–4 days after their birth) or around the third week of cellular age, we believe that the data presented in Figure 3B, C, E and F and in Figure 6B, C represent a net effect on neurogenesis.

However, based on reviewer's suggestion, we have quantified the total number of $tdTom^{+}$ new neurons present at day 30 in sections obtained from mice treated with IgG-Sap and p75-Sap and have included these additional data in the Figure 5 (Fig. 5D). These data complement those reported in the Figure 4 showing a significant reduction in the number of new neurons ($BrdU^{+}DCX^{+}$) in p75-Sap- compared to IgG-Sap-injected mice.

Reviewer 2 Advance Summary and Potential Significance to Field:
The authors build on previous results addressing the importance of the cholinergic input to the dentate gyrus in the regulation of adult hippocampal neurogenesis. The focus is on muscarinic receptor 4 (M4) and in vitro and in vivo approaches are employed to make the case that this receptor drives direct effects of cholinergic input on NPCs and newborn neurons of the hippocampal neurogenic niche.

Response to Reviewer 2:

The focus is on muscarinic receptor 4 (M4) and in vitro and in vivo approaches are employed to make the case that this receptor drives direct effects of cholinergic input on NPCs and newborn neurons of the hipocampal neurogenic niche. The variety of approaches and experimental setups is to be appreciated. However, the manuscript lacks a bit of focus and a variety of scattered experiments in vivo and in vitro could be traded for a more thorough and deeper analysis.

1. ***Importantly selective loss-of-function of the muscarinic receptors in specific cell types (NSCS, NPCs, immature neurons) are missing. Even if the expression of muscarinic receptors has been previously shown and the notion is reinforced herein by in vitro work, the results presented here cannot negate the possibility that the observed effects could be mediated indirectly by many other cell types in the niche. I understand that doing these experiments represents a major effort might be out of the scope of this work. At least, it should be crystal-clear what is known about the expression of the muscarinic receptors in the cell types of interest in vivo, from previous literature and available sequencing data if direct expression by unambiguous immunostaining or similar is not available.***

Please see response #1 to Reviewer 1.

© 2024. Published by The Company of Biologists under the terms of the Creative Commons Attribution License (https://creativecommons.org/licenses/by/4.0/).

2. As the authors write in the discussion: "Although a rapid rise in intracellular calcium levels in NPCs has been reported following the application of acetylcholine to hippocampal slices (Itou et al., 2011), whether acetylcholine directly regulates the activity of distinct subpopulations of hippocampal NPCs (Jhaveri et al., 2015) is currently unknown." Unfortunately, the current manuscript does not answer this question either.

We respectfully disagree with the reviewer. In this study, using purified populations of NPCs (Nestin-GFP^{+}/EGFR^{+}) in a clonal density assay (< 1 cell/well), we provide evidence that stimulation of M4 muscarinic receptors directly activates a quiescent population of hippocampal NPCs. As our previous work identified two distinct subpopulations of quiescent NPCs that can be activated by the neurotransmitter norepinephrine or by KCl (Jhaveri et al., *J. Neuroscience*, 2015), we further show that stimulation of M4 mAChRs activates the same subpopulation of hippocampal NPCs as that which is responsive to norepinephrine (Fig. 2B). We have clarified this in the revised manuscript.

3. As an example, statements such as "… by direct innervation and through M4 mAChRs" must be toned down. The word "directly" is used too much. And the same applies to "selective" as in "Selective stimulation of the M4 mAChR enhances adult hippocampal neurogenesis in Vivo" because is broad stimulation of all the cells with the receptor and it is important that it is not confused with a selective activation of NSPCs.

Please see the response to above. However, based on the reviewer's suggestion, we have toned down our statements as appropriate.

4. Another important caveat is that it is not clear whether the focus are the NSPCs (as shown by the title) or the newborn/immature neurons. In the ablation and ablation + stimulation experiments overall cell proliferation but no NSPCs activation was assessed in contrast to the previous experiments. The experiments all must be systematic and consistent, especially if claims about proliferation vs. survival/differentiation are to be made.

The key novel finding of this study is the demonstration of expression of M4 mAChRs by quiescent hippocampal NPCs, and the ability of M4 mAChR-selective potentiators to activate these cells, resulting in increased numbers of newborn neurons. Given the pro-neurogenic effects of the M4 mAChR potentiators, the rationale for the ablation experiment was to investigate whether impairments in hippocampal neurogenesis due to loss of cholinergic innervation could be rescued by activating NPCs via stimulation of mAChRs *in vivo*. This has been clarified in the manuscript on page 7.

5. In the in vitro experiments, it is not clear what populations are the authors referring to with Nestin-GFP+EGFR− cells vs Nestin- GFP+EGFR+ cells. What would be their in vivo counterparts? A more factual characterization of these populations in vitro is required given the results regarding CHRM4 expresion and to make a solid case. The case is similar for the KCL vs. norepinephrine. What is the addition of this experiment? What in vivo cell populations are these cells representing.

Nestin-GFP^{+}/EGFR^{+} cells represent a near-pure population of neurosphere-forming hippocampal NPCs which we have extensively characterised. This NPC population, which is isolated from the adult mouse hippocampus, comprises two subpopulations of quiescent NPCs, one which is activated by norepinephrine and one that is responsive to KCl (Jhaveri et al., *J. Neuroscience,* 2015). We have demonstrated the presence of these co-labelled cells in the subgranular zone *in vivo*, with 32.6 ± 7.9% of Nestin-GFP^{+}/EGFR^{+} cells exhibiting a radial glia-like (RGL) morphology and 67.4 ± 7.9% having a non-RGL morphology. Nestin-GFP^{+}/EGFR^{-} cells do not form neurospheres *in vitro*, even in response to stimulation by norepinephrine or KCl. To investigate whether stimulation of M4 mAChRs activated either or both subpopulations of quiescent NPCs, we conducted the neurosphere assay and examined the effects of VU10010, a selective allosteric potentiator of M4 mAChRs on the total neurosphere numbers in the presence of norepinephrine or KCl (Fig. 2B). These points have been clarified in the revised manuscript.

7. Related to this, the term "quiescent" should be used more carefully, no analysis of cell re-entry of first-time activation was done, so just avoid it when unnecessary.

© 2024. Published by The Company of Biologists under the terms of the Creative Commons Attribution License (https://creativecommons.org/licenses/by/4.0/).

We have defined our use of the term ‘quiescent’ NPCs in the revised manuscript. These are latent, non-proliferating NPCs *in vitro* and *in vivo* under baseline conditions and are prospectively identified following activation (for example, following stimulation of M4 mAChR) as those NPCs that exhibit cell proliferation *in vivo* (for example, BrdU incorporation) or neurosphere formation *in vitro*. We trust that this is acceptable to the reviewer.

8. It would be useful to at least check cell proliferation with any marker to confirm that cholinergic stimulation increases the number of neurospheres by promoting cell division.

In our study, neurospheres were generated by clonal expansion and proliferation of an individual NPC. Therefore, an increase in the number of neurospheres in response to cholinergic stimulation reflects the number of ‘proliferating’ NPCs. We have clarified this in the Results on page 5.

9. Also, in Fig. 1 it would be better to plot the control(s) with their SEM too and run the correspondent statistical tests for several groups.

Each neurosphere assay uses many animals and we do observe batch variation in the total number of neurospeheres obtained in the control (baseline condition). Hence, we have normalised the data from each biological replicate with respect to the control and plotted test groups as the percent change. We have then used the repeated measures ANOVA or paired t-tests to statistically test for differences between groups, which we believe is the most appropriate approach in this case.

10. In the ablation experiments it has to be explored and ruled out that neuronal death and degeneration is not affecting the neurogenic niche rather than just the absence of muscarinic innervation. Checking local neuroinflammation in the DG would help.

To investigate the effects of medial septum (MS) and diagonal band of Broca (DBB) cholinergic neuron loss on the structural integrity of the dentate gyrus (DG), we quantified this area in hippocampal sections from IgG-Sap- versus p75-Sap-treated mice. Our results revealed no difference between these groups in either C57Bl/6J or Ascl1-Cre^{ERT2}::tdTom mice. Data from C57Bl/6J mice have been included in Figure 4 (Fig. 4I).

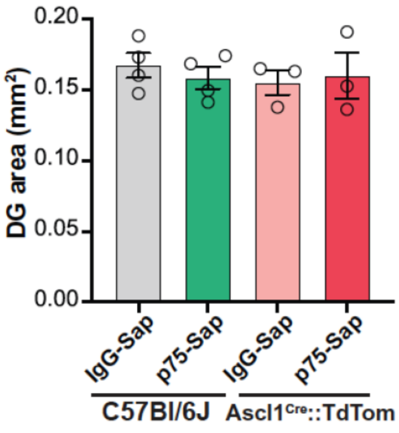

We also examined neuroinflammation by quantifying the number of Iba1$^+$ microglia in the DG of IgG-Sap- versus p75-Sap-treated mice and found no difference in their numbers between groups (see below). These data are included in Figure 4 (Fig. 4J, K). This is consistent with a previous study which also reported no significant changes in neuroinflammatory markers or microglial activity in the hippocampus of MS cholinergic neuron-lesioned mice when compared to the control at day 40 post-lesion (Field et al., *J. Neuroscience*, 2012).

© 2024. Published by The Company of Biologists under the terms of the Creative Commons Attribution License (https://creativecommons.org/licenses/by/4.0/).

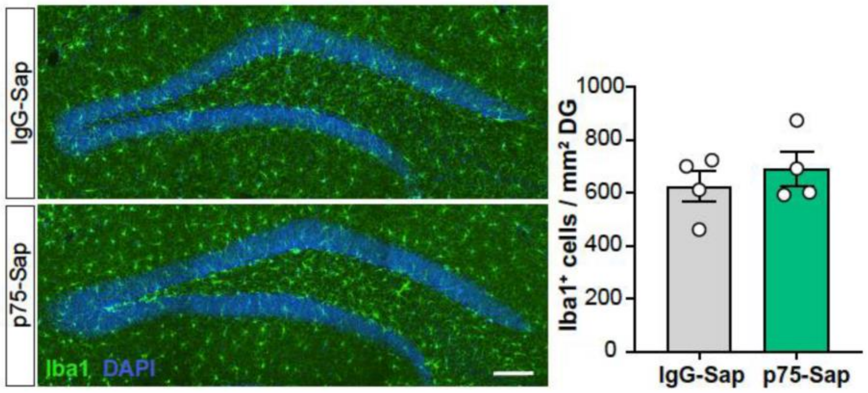

11. The specificity of the injection and subsequent ablation is convincing, but a better control, and more informative for the manuscript, would be to ablate cells in nearby nuclei (NBM and SI).

As the nucleus basalis of Meynert (NBM) and substantia innominata (SI) cholinergic neurons innervate the entire neocortex and the amygdala, ablation of these cholinergic neurons could impact hippocampal neurogenesis via indirect mechanisms. We are not convinced that this experiment would enhance our finding, and we trust that the reviewer will agree that it does not warrant the use of additional animals.

12. Figure 2B; C, E and F would be more informative showing total numbers rather than percentage. It can lead to confusion (BrdU+DCX+ percentage of BrdU+ or of DCX+…?). Also in Fig. 3K-N…

We respectfully disagree. As detailed in the methods section, quantification was performed using stereology and cell numbers in the dentate gyrus were divided by the length of the subgranular zone. Data have been expressed as 'percentage change' relative to the control and presented as mean ± SEM for each group to simplify the presentation.

13. All the "data not shown" data should be shown or not mentioned at all.

As these data do not impact the overall claims and conclusion of this study, we have chosen to remove them from the revised manuscript.

14. The title should reflect that what the papers shows is a variety of effects, direct or indirect on several processes of the neurogenic cascade.

We believe that the title highlights the key findings of our study. If a change is deemed necessary, we would appreciate suggestions.

Reviewer 3 Advance Summary and Potential Significance to Field:

Adult hippocampus neurogenesis has been established to be a key mechanism in hippocampal function. This process is impaired in aging and neurodegenerative disorders. Creating a better understanding of mechanisms regulating hippocampal neurogenesis remains a high priority area. While overall signaling is known to affect hippocampal neurogenesis, the contribution of MS and DBB neurons, two major populations of cholinergic neurons in the BF, and the identity of the cholinergic receptor subtypes remain to be established. This manuscript describes studies demonstrating that proliferation of quiescent, hippocampal NPCs is affected by MS and DBB neurons and that these effects are mediated, at least in part, through activation of M4 mAChRs. An unexpected finding was that administration of an M4-selective allosteric potentiator, in the context of cholinergic dysfunction, impaired dendritic morphological maturation of newly born hippocampal neurons. This study makes a key set of contributions to our understanding of basal forebrain cholinergic neuron regulation of fundamental areas of hippocampal neurogenesis and

© 2024. Published by The Company of Biologists under the terms of the Creative Commons Attribution License (https://creativecommons.org/licenses/by/4.0/).

will have a broad contributor impact for this field. The paper is well written, and the methods are clearly presented. The statistical approaches seem to be appropriate.

Reviewer 3 Comments for the Author:

1. In the Introduction it would be helpful to clarify what is known about M4 receptor expression by NPCs or whether this study is the first to demonstrate that NPCs expression the M4 subtype.

As suggested, we have now included the point about the expression of M4 receptors in adult hippocampal NPCs in the Introduction.

2. In the first paragraph of the Results, it would be helpful to define "activity" of NPCs as meaning proliferation. In this paragraph, the point is made that it is not known if acetylcholine directly affects proliferation of NPCs, yet in the Introduction the observation is cited that stimulation of M1 receptors promote NPC proliferation; this is a bit confusing. Perhaps the current state of knowledge on effects of targeting cholinergic receptors on NPCs could be better clarified.

Thank you for this suggestion; we have now added an explanation of the term "activity" which indicates the proliferative capacity of NPCs when cultured *in vitro* in the Results. We have also clarified the current knowledge on the expression and function of cholinergic receptors on NPCs in the Introduction.

3. Treatment of NPCs with 50 uM muscarine. Is this a traditional concentration? Is it physiologically relevant and/or in line with its Kd? This seems like a high concentration.

The typical dose of muscarine used in physiological brain slices studies ranges between 10 µM– 50 µM and is dependent on the amplitude of the depolarising pulse administered to the nerve terminal as well as the brain region examined. (Slutsky et al., *J. Physiology*, 1999; Scroggs et al., *J. Neurophysiology*, 2001). Notably, in our study, muscarine (10 µM or 50 µM) was added only once at the start of NPCs culture, and neurospheres were quantified at day14.

4. Page 6, second paragraph, NPCs were plated at "clonal density". It would be helpful to know what clonal density is and the rational for its use.

We have clarified the term clonal density (<1 cell per well) and the rationale for its use. Performing the neurosphere assay by culturing NPCs at this density enabled us to probe direct effects of stimulating M4 mAChRs (in the absence of any niche cells) on the regulation of hippocampal NPC proliferation and subsequent neurosphere formation.

5. The combination of the in vitro and in vivo studies is a strong point. The finding that loss of cholinergic innervation to the hippocampus affects survival of newly generated progenitors or neurons more than baseline proliferation or neurogenic differentiation is a key finding.

We thank the reviewer for the positive comment.

6. Page 11, Discussion, top paragraph. Is the present study the first to show, directly using NPCs, that cholinergic receptor stimulation, of any form, can promote NPC proliferation or neurogenesis?

Yes, the present study is the first to report the effects of stimulating muscarinic cholinergic receptors, specifically M4 mAChRs, in promoting NPC proliferation *in vitro* and *in vivo* and enhancing neurogenesis *in vivo*. We have included this point in the discussion.

7. A valuable part of the Discussion with broad implication is that strategies promoting survival or functions of MS/DBB cholinergic neurons could serve to maintain ongoing neurogenesis in AD and other pathological settings. Another very valuable point is that suggestion that any targeting of M4 related mechanisms must examine structural and

© 2024. Published by The Company of Biologists under the terms of the Creative Commons Attribution License (https://creativecommons.org/licenses/by/4.0/).

functional aspects of NPCs in addition to traditional measures of proliferation or neurogenesis.

We thank the reviewer for the positive comment.

8. Are any cognitive effects in the various mouse models of M4 potentiators known?

The discovery of highly selective M4 positive allosteric modulators (aka potentiators), including those used in the present study (VU10010 and VU0152100), has provided important insights into the roles of this receptor in regulating cognitive and behavioural outcomes in preclinical models of multiple central nervous system disorders such as schizophrenia, substance abuse disorders and Alzheimer's disease (for in-depth reviews, see Foster et al., *Neuropsychiatric Disease and Treatment*, 2014; Foster and Conn, *Neuron*, 2017). For example, there is a growing literature showing an antipsychotic drug-like profile for VU0152100 (Brady et al., *J Pharmacol Exp Ther*. 2008; Byun et al., *Neuropsychopharmacology*, 2014). There is also evidence supporting the beneficial effects of M4 potentiators in preclinical models of associative learning and memory functions (Bubser et al., *ACS Chemical Neuroscience*, 2014).

We have now included this information in the revised Discussion on pages 13-14.

9. Figures. Overall, the figures are well presented. For Figure 1C, it would be helpful to better describe the measures in the Y axis.

We have clarified the measure FPKM (Fragments Per Kilobase of transcript per Million mapped reads) in the figure legend for Fig. 1C. It is an expression level method used for RNA-seq data to normalise the read count based on gene length and the total number of mapped reads.

<u>Second decision letter</u>

MS ID#: DEVELOP/2023/201835

MS TITLE: Stimulation of the muscarinic receptor M4 activates quiescent neural precursor cells and partially ameliorates medial septum cholinergic lesion-induced impairments in adult hippocampal neurogenesis

AUTHORS: Lidia Madrid, Katelyn Hafey, Saurabh Bandhavkar, Gabriela O Bodea, Javier Jimenez-Martin, Michael Milne, Tara L. Walker, Geoffrey Faulkner, Elizabeth J Coulson, and Dhanisha J Jhaveri

I have now received the reports of two of the referees who reviewed the 1st version of your manuscript and I have reached a decision. The referees' comments are appended below, or you can access them online: please go to BenchPressand click on the 'Manuscripts with Decisions' queue in the Author Area.

As you will see, the two referees retain a great interest in your work, but they still have significant criticisms and recommend a further substantial revision of your manuscript before we can consider publication. In particular, they criticise the interpretation of the data on M4 mAChR agonists promoting neural precursor cell proliferation and emphasise that there is no evidence that these agonists act by activating quiescent NPCs rather than by promoting the proliferation of already dividing NPCs. They also disagree with the conclusion that M4mAChR is expressed on quiescent NPCs. Referee 2 also contests the conclusion that a M4 agonist treatment partially rescues or ameliorates a MS/DBB-lesion induced decrease in adult-born neurons since the difference in numbers of BrdU+ DCX+ cells obtained after vehicle and VU treatments are not significant (p =3D 0.082 in Fig. 6B).

Having carefully read the revised manuscript, I must say that I agree with the referees' comments. It seems that your interpretation that M4 mAChRs are expressed by and act on quiescent NPCs is

© 2024. Published by The Company of Biologists under the terms of the Creative Commons Attribution License (https://creativecommons.org/licenses/by/4.0/).

based on the assumption that purified neurosphere-forming Nestin-GFP+ EGFR+ cells are quiescent. However I haven't found evidence for this in your manuscript or in the literature. The increase in number of neurospheres upon M4 agonist treatment of Nestin-GFP+ EGFR+ cells could be due to an increase in the survival or in the proliferation rate of already dividing NPCs. Proliferative NPCs cultivated in clonal conditions could either die or divide at a rate too low to generate neurospheres in control conditions. M4 agonists could allow these cells to survive or could increase their proliferation rate so that they are able to generate neurospheres. The experiments performed in this study do not distinguish between these possibilities and a direct activation of quiescent NPCs.

Furthermore, your conclusion that M4 agonists promote the activation of quiescent NPCs in vivo is based on the assumption that NPCs exhibiting an RGL morphology are quiescent (last paragraph of p. 6). However this statement is contradicted by numerous published studies and by your own data in Fig. 2G showing that a small but significant fraction of RGLs (4% in your figure) incorporate BrdU and are therefore cycling and not quiescent. The increase in the fraction of BrdU+ RGLs could be due to an increased proliferation of already active RGLs rather than by an activation of quiescent RGL cells. Again your analysis does not allow to distinguish between these different possibilities.

If you are able to revise the manuscript along the lines suggested, I will be happy receive a revised version of the manuscript. In the new revised version, you should leave open the different mechanisms by which M4 agonists might stimulate NPCs proliferation in vitro and in vivo, and you should remove the conclusion that M4 agonists partially rescue a MS/DBB-lesion-induced loss of new neurons. Your revised paper will be re-reviewed by the original referees, and its acceptance will depend on your addressing satisfactorily all their major concerns.

If it would be helpful, you are welcome to contact us to discuss your revision in greater detail. Please send us a point-by-point response indicating your plans for addressing the referees' comments, and we will look over this and provide further guidance.

Please attend to all of the reviewers' comments and ensure that you clearly highlight all changes made in the revised manuscript. Please avoid using 'Tracked changes' in Word files as these are lost in PDF conversion. I should be grateful if you would also provide a point-by-point response detailing how you have dealt with the points raised by the reviewers in the 'Response to Reviewers' box. If you do not agree with any of their criticisms or suggestions please explain clearly why this is so.

Reviewer 1

Advance summary and potential significance to field

The authors added novel data to address our previous concerns. However, we are still not fully convinced by the interpretation of their data.

Comments for the author

We had suggested to provide direct in vivo evidence for expression of Chrm4 in quiescent hippocampal stem cells and to provide additional in vitro evidence that Chrm4 regulates quiescence of hippocampal progenitors.
The authors nicely added a new set of RNA-scope data. However, it remains unclear if Chrm4 is indeed expressed in quiescent cells: one nestin-expressing, radial glia-like (RGL) cell is shown; however, these cells are not strictly quiescent but can divide (indeed, it seems that in their hands many RGLs divide (see Fig. 2 H vs 2I) - the fraction of Chrm4 expression in non-dividing vs. dividing progenitor cells remains unclear). Thus, additional quantifications and phenotyping of Chrm4 cells may be helpful.

Indeed, most (if not all) in vivo, single cell RNA-sequencing data suggest that Chrm4 is not detectable in quiescent NSCs isolated from the adult hippocampus. We understand that the authors find enrichment of Chrm4 in NesGFP/EGFR+ cells; however, it is not clear (or fully accepted) that these cells contain a bona fide quiescent population (rather, one my argue that those are "activated" progenitor cells and therefore are efficient to generate neurospheres). This is line with the attempts of the authors to use BMP-induced quiescence of progenitors where no Chrm4

© 2024. Published by The Company of Biologists under the terms of the Creative Commons Attribution License (https://creativecommons.org/licenses/by/4.0/).

expression was observed – again, in line with most of the available in vivo scRNA-seq data sets (we fully agree that the BMP-mediated model does not necessarily reflect the in vivo state of quiescent cells...).

Thus, we are somewhat concerned with the interpretation of the data (the data by themselves are concinving with regards of cellular phenotypes upon M4 manipulations): is it really true that Chrm4 is expressed in quiescent hippocampal NSCs and that the observed effects are based on alterations in cellular quiescence? Or is it rather true that Chrm4 becomes upregulated in activated NSCs and that expansion/proliferation is, for example, boosted by muscarinic stimulation? Indeed, we are afraid that with the currently available data a distinction between these two possibilities is rather difficult to make (even though we would argue that the in vivo mRNA expression data rather suggest that M4 activation affects active progenitor cell divisions; but not bona fide quiescent cells). Thus, we may suggest that the authors (substantially) modify the interpretation of their data and the conclusions drawn.

<u>Reviewer 2</u>

Advance summary and potential significance to field

The experimental effort for the revision has been minimal, addressing only point 1 of referees 1 and 2.

The only real claims that the authors can make are: 1, that M4 receptors are expressed in a subpopulation of NSPCs and that the activation of these M4 receptors promotes their proliferation; and 2, that inducing the loss of cholinergic neurons decreases newborn neuron survival and alters dendritic morphology.

The use of an allosteric potentiator of M4 receptors in an attempt to restore neurogenesis does not work in spite of the author´s claims. There are no differences between the groups (Fig. 6 B and C).

Overall, if no more experimental effort is to be made, the text must be thoroughly revised to be adjusted to the information provided by the data, but the relevance of the work will be compromised.

Comments for the author

The new experiments do sustain the expression of M4 in NPCs, but the question is also about what other cell types (astrocytes, granule cells and importantly newborn neurons) in the niche are expressing them too. This has to be clearly stated in the introduction and discussed because of the probable indirect effects as acknowledged by the authors. Of special interest is the expression of cholinergic receptors by newborn neurons, given the effect on survival and dendritic morphology found after the lesion of the MS/DBB areas. As the results show (Figure 1D) there is extensive M4 mRNA staining in the neurogenic niche.

The use of an allosteric potentiator of M4 receptors to compensate the loss of neurogenesis induced by loss of cholinergic neurons neurogenesis fails. There is no difference between the lesioned mice with vehicle and the lesioned mice treated with the potentiator in terms of newborn neurons (Fig. 6 B and C) and the dendritic alterations are worsened. The claims that the authors make in the title and text are therefore not supported by the data, for the "partially ameliorates medial septum cholinergic lesion-induced impairments in adult hippocampal neurogenesis" part. While the expression of M4 and effect on proliferation is clear, the rest of the work, that is, the loss of cholinergic input (regardless of receptors) effect on the dendritic morphology of newborn neurons and absence of effect on proliferation and failure to restore neurogenesis is weaker.

It Is not clear whether this is an indirect effect or a direct one, or a mix.
There´s no information about the cholinergic receptors expressed by newborn neurons if any and what could be the mechanisms governing the effects on dendritic arborization.

© 2024. Published by The Company of Biologists under the terms of the Creative Commons Attribution License (https://creativecommons.org/licenses/by/4.0/).

Also, the authors insist in using the term “quiescent” or even “latent” and I think is not appropriate at all. There is not a single experiment in the work devised to distinguish between recruitment and activation of quiescent NSCs/NPCs or just inducing additional round of division in already activated ones. This per se does not invalidate the results on NSC/NPC proliferation but the text must exclude any possible confusion for the audience and I do not see the reason to stretch the conclusions that far.

Proliferation is induced, but the manuscript does not really investigate whether the proliferating cells were really quiescent or within the round of several mitosis that NPCs undergo. In fact, quiescent is a term really used for NSCs rather than for NPCs. The previous bibliography extensively shows that NPCs divide several times consecutively before maturing into neurons. The manuscript does not show whether NPCs divide more times rather than exit quiescence. In reality latency or quiescence are not really tested.

“including those exhibiting radial glial-like (RGL) morphology which are considered to be quiescent NPCs” Radial-like Nestin-GFP cell comprise both quiescent and activated NSPCs, specially, if only Nestin-GFP+EGFR+ are selected it is more likely that they are in a primed or preactivated state according to the bibliography. The EGFR- population could be considered to be quiescent.
It is important to clarify that Nestin-GFP+EGFR+ and Nestin-GFP+EGFR- can both include NSCs and NPCs. On the other hand, it should be discussed what those norepinephrine-, or KCl-responsive subpopulations could be in vivo because other ways the contribution of those experiments is not clear.

Second revision

Author response to reviewers' comments

Response to Reviewers - MS ID#: DEVELOP/2023/201835

We thank the editor reviewers for their constructive criticism and questions, which we have addressed below.

Response to Reviewer 1

Reviewer 1 Advance Summary and Potential Significance to Field:

The authors added novel data to address our previous concerns. However, we are still not fully convinced by the interpretation of their data.

1. We had suggested to provide direct in vivo evidence for expression of Chrm4 in quiescent hippocampal stem cells and to provide additional in vitro evidence that Chrm4 regulates quiescence of hippocampal progenitors. The authors nicely added a new set of RNA-scope data. However, it remains unclear if Chrm4 is indeed expressed in quiescent cells: one nestin-expressing, radial glia-like (RGL) cell is shown; however, these cells are not strictly quiescent but can divide (indeed, it seems that in their hands many RGLs divide (see Fig. 2 H vs 2I) - the fraction of Chrm4 expression in non-dividing vs. dividing progenitor cells remains unclear). Thus, additional quantifications and phenotyping of Chrm4 cells may be helpful.

We have conducted additional analysis on sections used for single-molecule *in situ* hybridization and have quantified the proportion of Nestin-GFP$^+$ cells expressing Chrm4 transcripts. We found that 6.4 ± 1.8% of the total Nestin-GFP$^+$ cells express Chrm4, which is similar to the Nestin-GFP$^+$/EGFR$^+$ NPC population which constitutes 4.7 ± 1.0% of the total Nestin-GFP cells (Jhaveri et al., *J. Neuroscience*, 2015). We have added these additional data to Figure 1. Whether and what fraction of these Nestin-GFP$^+$/Chrm4$^+$ NPCs are dividing currently remains uncharacterized. We have therefore modified our interpretation regarding the effects of M4 stimulation on NPC activity

© 2024. Published by The Company of Biologists under the terms of the Creative Commons Attribution License (https://creativecommons.org/licenses/by/4.0/).

and have left open the possibility that these may occur either via activation of quiescent NPCs or by enhancing the proliferation of already active NPCs in the adult hippocampus.

2. Indeed, most (if not all) in vivo, single cell RNA-sequencing data suggest that Chrm4 is not detectable in quiescent NSCs isolated from the adult hippocampus. We understand that the authors find enrichment of Chrm4 in NesGFP/EGFR+ cells; however, it is not clear (or fully accepted) that these cells contain a bona fide quiescent population (rather, one my argue that those are "activated" progenitor cells and therefore are efficient to generate neurospheres). This is line with the attempts of the authors to use BMP-induced quiescence of progenitors where no Chrm4 expression was observed - again, in line with most of the available in vivo scRNA-seq data sets (we fully agree that the BMP-mediated model does not necessarily reflect the in vivo state of quiescent cells...). Thus, we are somewhat concerned with the interpretation of the data (the data by themselves are convincing with regards of cellular phenotypes upon M4 manipulations): is it really true that Chrm4 is expressed in quiescent hippocampal NSCs and that the observed effects are based on alterations in cellular quiescence? Or is it rather true that Chrm4 becomes upregulated in activated NSCs and that expansion/proliferation is, for example, boosted by muscarinic stimulation? Indeed, we are afraid that with the currently available data a distinction between these two possibilities is rather difficult to make (even though we would argue that the in vivo mRNA expression data rather suggest that M4 activation affects active progenitor cell divisions; but not bona fide quiescent cells). Thus, we may suggest that the authors (substantially) modify the interpretation of their data and the conclusions drawn.

Based on the reviewer's suggestion, we have modified our statements regarding the cellular state of the M4 mAChR-expressing hippocampal NPCs throughout the manuscript by not referring them as quiescent NPCs. We have also included an additional paragraph in the discussion (page 11) considering potential mechanisms by which M4 mAChRs may enhance NPC activity and boost adult hippocampal neurogenesis.

Response to Reviewer 2

Reviewer 2 Advance Summary and Potential Significance to Field:

The experimental effort for the revision has been minimal, addressing only point 1 of referees 1 and 2.

We respectfully disagree with this comment. In addition to conducting qPCR and RNAScope experiments to confirm the expression of M4 mAChRs in the hippocampal NPCs (point 1 of reviewers 1 and 2), we also conducted an experiment using an *in vitro* model of quiescence. However, as we found that these cells had no or very negligible expression of *CHRM4,* the stimulation experiment was untenable. Furthermore, based on this reviewer's comment, we also examined neuroinflammation by quantifying the number of Iba1^{+} microglia in the dentate gyrus. These additional data were included in Figure 4 (Fig. 4J, K) during the last revision.

The only real claims that the authors can make are: 1, that M4 receptors are expressed in a subpopulation of NSPCs and that the activation of these M4 receptors promotes their proliferation; and 2, that inducing the loss of cholinergic neurons decreases newborn neuron survival and alters dendritic morphology. The use of an allosteric potentiator of M4 receptors in an attempt to restore neurogenesis does not work in spite of the author´s claims. There are nodifferences between the groups (Fig. 6 B and C). Overall, if no more experimental effort is to be made, the text must be thoroughly revised to be adjusted to the information provided by the data, but the relevance of the work will be compromised.

1. The new experiments do sustain the expression of M4 in NPCs, but the question is also about what other cell types (astrocytes, granule cells and importantly newborn neurons) in the niche are expressing them too. This has to be clearly stated in the introduction and discussed because of the probable indirect effects as acknowledged by the authors. Of special interest is the expression of cholinergic receptors by newborn neurons, given the effect on

© 2024. Published by The Company of Biologists under the terms of the Creative Commons Attribution License (https://creativecommons.org/licenses/by/4.0/).

survival and dendritic morphology found after the lesion of the MS/DBB areas. As the results show (Figure 1D) there is extensive M4 mRNA staining in the neurogenic niche.

Data regarding the precise expression pattern of M4 mAChRs in various hippocampus cell types is not currently available. However, the Allen Brain Atlas as well as previous studies (Levey et al., *J. Neurosci*., 1995; Rouse and Levey., *JCN*, 1996) show that M4 mAChRs are differentially expressed across hippocampal sub-regions. Whether M4 mAChRs are expressed by hippocampal adult-born neurons during their immature phase remains unknown but is an active area of investigation within our laboratory. As we feel this is an interesting point for Discussion, we have included it in this section (page 13), rather than in the Introduction.

2. The use of an allosteric potentiator of M4 receptors to compensate the loss of neurogenesis induced by loss of cholinergic neurons neurogenesis fails. There is no difference between the lesioned mice with vehicle and the lesioned mice treated with the potentiator in terms of newborn neurons (Fig. 6 B and C) and the dendritic alterations are worsened. The claims that the authors make in the title and text are therefore not supported by the data, for the "partially ameliorates medial septum cholinergic lesion-induced impairments in adult hippocampal neurogenesis" part. While the expression of M4 and effect on proliferation is clear, the rest of the work, that is, the loss of cholinergic input (regardless of receptors) effect on the dendritic morphology of newborn neurons and absence of effect on proliferation and failure to restore neurogenesis is weaker.

Although we observed a strong trend towards an increase in the number of newborn neurons ($BrdU^{+}DCX^{+}$) in the p75-Sap+VU0152100-treated mice compared to the p75-Sap+vehicle group, we agree with the reviewer that the pharmacological stimulation of M4 mAChRs could not fully compensate for the MS/DBB cholinergic lesion-induced reduction in hippocampal neurogenesis. We have therefore modified our interpretation and conclusion in the Results and Discussion.

3. It not clear whether this is an indirect effect or a direct one, or a mix. There´s no information about the cholinergic receptors expressed by newborn neurons, if any, and what could be the mechanisms governing the effects on dendritic arborization.

There are no published studies reporting the expression of M4 mAChRs in newborn neurons. However, we are currently investigating the transcriptome of 4 week-old newborn hippocampal neurons. How septal cholinergic neurons regulate the morphological development of adult-born neurons in the hippocampus remains an interesting and important question for future research which is beyond the scope of the current study. We have discussed likely direct and indirect mechanisms underpinning this effect in our Discussion on page 13.

4. Also, the authors insist on using the term "quiescent" or even "latent" and I think is not appropriate at all. There is not a single experiment in the work devised to distinguish between recruitment and activation of quiescent NSCs/NPCs or just inducing additional round of division in already activated ones. This per se does not invalidate the results on NSC/NPC proliferation but the text must exclude any possible confusion for the audience and I do not see the reason to stretch the conclusions that far. Proliferation is induced, but the manuscript does not really investigate whether the proliferating cells were really quiescent or within the round of several mitosis that NPCs undergo. In fact, quiescent is a term really used for NSCs rather than for NPCs. The previous bibliography extensively shows that NPCs divide several times consecutively before maturing into neurons. The manuscript does not show whether NPCs divide more times rather than exit quiescence. In reality latency or quiescence are not really tested.

We have removed the use of the term 'quiescence' through most of the manuscript. We have modified our interpretation regarding the effects of M4 stimulation on NPC activity and have left open the possibility that these may occur either via activation of quiescent NPCs or by enhancing the proliferation of already active NPCs in the adult hippocampus.

5. Radial-like Nestin-GFP cell comprise both quiescent and activated NSPCs, specially, if only Nestin-GFP+EGFR+ are selected it is more likely that they are in a primed or preactivated state according to the bibliography. The EGFR- population could be considered to be

© 2024. Published by The Company of Biologists under the terms of the Creative Commons Attribution License (https://creativecommons.org/licenses/by/4.0/).

quiescent. It is important to clarify that Nestin-GFP+EGFR+ and Nestin-GFP+EGFR- can both include NSCs and NPCs.

We agree with the reviewer. In fact, in our previous study (Jhaveri et al., *J. Neuroscience,* 2015), we extensively discussed the functional state of Nestin-GFP$^+$/EGFR$^+$ cells and based on our findings suggested that the expression of EGFR alone is not sufficient to distinguish between active and quiescent hippocampal precursor cells. We have proposed transactivation of EGFR signaling by G-protein-coupled receptor (GPCR) family such as β3-adrenergic receptors (Jhaveri et al., *J. Neuroscience,* 2010) or KCl acting via L-type calcium channels as a mechanism that would ensure the rapid cell cycle entry of activatable (EGFR$^+$) precursor cells upon neurogenic stimulation while preserving the non-activatable or dormant (EGFR$^-$) precursor pool. We have added this point to our Discussion on page 11.

6. On the other hand, it should be discussed what those norepinephrine-, or KCl-responsive subpopulations could be in vivo because other ways the contribution of those experiments is not clear.

We have previously demonstrated the presence of distinct subpopulations of activatable, self-renewing and multipotent NPCs that are responsive to either KCl or norepinephrine (Jhaveri et al., 2010; Walker et al., 2008). Our studies have found that these NPC subpopulations are not only differentially distributed along the septotemporal axis of the adult hippocampus but are also differentially regulated by neurogenic modulators such as GABA, corticosterone, and selenium (Jhaveri et al., 2015; Leiter et al., 2022). We have now included this information in the Results on page 6.

Third decision letter

MS ID#: DEVELOP/2023/201835

MS TITLE: Stimulation of the muscarinic receptor M4 regulates neural precursor cell proliferation and promotes adult hippocampal neurogenesis

AUTHORS: Lidia Madrid, Katelyn Hafey, Saurabh Bandhavkar, Gabriela O Bodea, Javier Jimenez-Martin, Michael Milne, Tara L. Walker, Geoffrey Faulkner, Elizabeth J Coulson, and Dhanisha J Jhaveri
ARTICLE TYPE: Research Article

I am delighted to tell you that your manuscript has been accepted for publication in Development, pending our standard ethics checks.

© 2024. Published by The Company of Biologists under the terms of the Creative Commons Attribution License (https://creativecommons.org/licenses/by/4.0/).
